# Physico-Mechanical Performances of Mortars Prepared with Sorted Earthquake Rubble: The Role of CDW Type and Contained Crystalline Phases

**DOI:** 10.3390/ma16072855

**Published:** 2023-04-03

**Authors:** Antonio Galderisi, Miguel Bravo, Gianluca Iezzi, Giuseppe Cruciani, Eleonora Paris, Jorge de Brito

**Affiliations:** 1 Dipartimento INGEO (Ingegneria & Geologia), Università di Chieti-Pescara ‘G. d’Annunzio’, 66100 Chieti, Italy; 2 CERIS, Instituto Superior Técnico, Universidade de Lisboa, 1049-001 Lisboa, Portugal; 3 Istituto Nazionale di Geofisica e Vulcanologia—INGV, 00143 Rome, Italy; 4 Dipartimento di Fisica e Scienze della Terra, Università di Ferrara, 44122 Ferrara, Italy; 5 Scuola di Scienze e Tecnologie, sez. Geologia, Università di Camerino, 62032 Camerino, Italy

**Keywords:** CDW, XRPD, mechanical properties, recycled aggregate, mortar

## Abstract

Construction and demolition waste (CDW) from earthquake rubbles was used here as recycled aggregates (RA) in cementitious binders. The materials were sorted in six groups: concrete (CO), natural stone (NS), tile (TI), brick (BR), perforated brick (PF) and roof tile (RT). The abundance (wt.%) of crystalline phases in each RA type was determined by X-ray Powder Diffraction (XRPD). Each group of RAs was used alone (100 wt.% of RA) and mixed with quartz-rich virgin aggregates (VA) to prepare 13 types of mortars (12 specimens per type): one reference mortar (RM) with only VA, six recycled aggregate mortars (RAM) and six recycled-plus-virgin aggregate mortars (RVAM). The physical and mechanical properties of aggregates and mortars reflect the type and abundance of crystalline phases in each CDW group. Recycled mortars rich in concrete, natural stones and tiles have better mechanical performance than mortars prepared with recycled bricks, perforated bricks and roof tiles. For each RA, RVAMs have superior mechanical characteristics than the corresponding RAM. Since the type and amount of phases contained in recycled aggregates strongly control the mechanical performance of new construction materials, they should be routinely quantified as reported here, in addition to other physical features (water absorption, density, etc.). The separation of heterogeneous CDW into homogeneous RA groups is necessary for the production of new construction materials with stable and predictable performances to ensure CDW recycling, especially in areas hit by major adverse events, where large amounts of still valuable materials could be used for reconstruction processes.

## 1. Introduction

Construction and demolition waste is the non-hazardous solid waste derived from construction, renovation and/or dismantling (but also destruction produced by wars) of buildings, as well as from natural catastrophic events such as earthquakes, landslides, flooding and/or volcanic eruptions. CDWs are mainly composed of ceramic- or mineral-like inert materials such as concrete, mortars, cements, masonries (tiles, roof-tiles and/or bricks) and building/ornamental stones [1]. In addition, variable but generally minor amounts of metals, plastics, textiles, wood, glass, waste of electric and electronic equipment (RAEE) occur in CDW. Asphalts, soil and/or dredging materials are often preliminarily separated and eventually recycled, as in the case of asphalts [2,3].

In Europe, CDW corresponds to about one third of the total waste generated [1]. A similar estimation holds for all the other countries and continents. These huge amounts of end-of-life (EoL) waste require its significant recycling and reusing, limiting further landfilling and consequent extraction of new and virgin natural materials for the production of aggregates, i.e., virgin aggregates (VA), cements and/or masonry [1]. The European Community recently imposed a CDW recycling target ≥70% by 2020; nevertheless, the actual recycling ratio is still variable between different EU states [4,5], and CDW is mostly used for downcycling applications.

The main problem of recycling and especially reusing CDW as secondary raw materials for new building products (e.g., concrete) is attributable to its heterogeneous features [6]. The heterogeneity of CDW strongly contrasts with the classical homogeneity of raw materials used to prepare construction materials. Certainly, the chemical, mineralogical, petrographic and textural variabilities in time and space of CDW prevents a determination and prediction of their physico-mechanical properties [4,7,8,9,10,11]. In fact, within the inert fraction of the CDW, diverse classes of materials with different physico-mechanical, petrographic and mineralogic features can be singled out, e.g., concrete, building/ornamental stone, brick, perforated brick, roof tile and tile [4,12,13,14,15,16,17,18].

Natural stones and concrete materials display the highest mineralogical and petrographic variability, depending on the geology of the area (affecting the lithotypes availability), and the traditional architectural style(s) [6]. In turn, natural stones and concrete mainly determine the heterogeneity of a CDW [6]. On the other hand, tiles and bricks, e.g., masonry, are relatively less heterogeneous in comparison, since they are prepared with materials rich in clay minerals [4,6,12,13,18]. Concrete is made of aggregates, cement and water in order of abundance and results from the hardening processes of this initially liquid-like mixes. After 28 days, concrete reaches around 60–90% of its final strength, and its mechanical properties are determined by the microstructure formed during hardening, mainly by the hydration reactions between cement and aggregates. Therefore, aggregates are fundamental in contributing to the strength of concrete and in controlling its dimensional variation, as well as in reducing production costs [19]. Virgin aggregates are low cost-per-ton materials and, consequently, they are mainly extracted locally, near the construction area, to limit transport charges. Consequently, their nature reflects the available lithotypes in source regions [4,6,12,16]. These aspects contribute to the heterogeneity of CDW and the consequential low value of these materials, which are commonly used in road foundations, foundation slabs and cavity back-fillings, hence for down-cycling applications [6,9,20,21,22].

The physico-mechanical properties of recycled aggregate concrete (RAC) are dependent on the type of CDW used as recycled aggregates (RA) [11,13,23,24,25,26,27]. For instance, RAs that are made mainly of concrete [28,29,30], ceramics/masonry [7,31,32,33,34,35,36], ceramics/tiles and sanitary wares [37,38,39] and glasses [40,41,42] show a different mechanical performance. In addition, when the amount of RA is low and VA prevails, the performance of RAC is relatively high [27,29,30,43]. All these aspects are even more important in areas hit by earthquakes, where the heterogeneity of CDW is higher due to buildings’ uncontrolled collapses. The post-disaster recovery of such areas can be significantly enhanced by the possible improved recycling of CDW rubbles, also aimed at reducing the environmental impact of CDW storage or landfilling.

This study focuses on the experimental assessment of physical and mechanical differences of laboratory-made mortars, obtained with the same cementitious binder but using different CDW fractions. The CDW rubbles were collected directly from the epicentre area of the 2016/2017 earthquakes, in the area surrounding the Amatrice city in Central Italy (Figure S1). They were manually separated to produce homogeneous materials enriched in natural stones (NS), concrete (CO), tiles (TI), bricks (BR), perforated bricks (PF) and roof tiles (RT). Then, they were used to prepare both recycled aggregate mortar (RAM) and recycled-plus-virgin aggregate mortar (RVAM). A reference mortar made of only VA was also prepared for comparison (see Figure S2 for a general reappraisal). While few other studies used X-ray Powder Diffraction (XRPD) to determine the mineralogical composition of RA [4,6,12,13,14,15], none of them performed the modal quantitative phase analysis (QPA). The main novelty of this study is that the QPA-Rietveld method was used to quantify, accurately, crystalline phases for the first time in CDW materials. Differently from previous qualitative determinations, our quantitative analysis of mineralogical, petrographic and physico-mechanical characterisations of the RA rubbles, VA and the resulting mortars, show the criteria that could be used for selecting the most useful CDW fractions for the production of new construction materials with the best performance. The outcomes of this work also provide a valuable appraisal for optimizing the sorting procedures of extremely heterogeneous CDW, such as earthquake rubbles, contributing to the resilience of an area affected by natural (flooding, etc.), and social or war disasters, as well as in the normal conditions of CDW streams’ production.

## 2. Previous Studies

In the literature, a huge number of studies (as detailed in the following) were devoted to investigating the behaviour of the mechanical properties of concrete (RAC) produced with recycled aggregates. These works focused on RA made of both concrete and masonry types, frequently mixed with VA in different proportions; however, the types of crystalline and non-crystalline phases in CDW are very rarely determined [6], while their amount was never measured in detail. In fact, to the best of the authors’ knowledge, no application of the quantitative phase analysis by the Rietveld method (QPA-Rietveld) has been ever performed on CDW. In turn, mechanical performances of construction materials prepared with CDW cannot be quantitatively related to their constitutive phases, given the lack of quantitative information.

The compressive and tensile strengths, as well as the modulus of elasticity, are the most important, and they represent common properties evaluated for assessing the mechanical performance. Kou et al. [23] produced RACs with different replacing fractions of RA made of concrete (RCA: recycled concrete aggregates) with respect to VA. They concluded that the negative influence of the use of the RCA they used is significant: the degradation of compressive and tensile strengths, as well as modulus of elasticity, is about 30–40% in RAC with respect to concrete made of VA only. Again, the absence of a quantitative determination of phases composing the aggregates does not allow direct comparisons among these RACs.

Etxeberria et al. [29] showed that, to obtain concrete with conventional compressive strength using coarse RCA, it was necessary to use a higher cement content. Similarly, Oliveira et al. [28] found that to produce a mix with 100% of coarse RCA with the same strength as standard concrete, it was necessary to increase the cement content of more than 15 wt.%. Poon et al. [24] observed that full replacement of the coarse VA with RCA from a recycling plant led to compressive strength losses of about 20–25% at 7 days and 10% at 90 days. Etxeberria et al. [29] observed that, when VA aggregates were substituted with 25 wt.% of coarse RCA, the degradation in compressive strength was negligible, whereas when the VAs were fully replaced with RCA, a 25% reduction in compressive strength resulted; tensile strength and modulus of elasticity showed a similar behaviour. It is thus possible to summarise, qualitatively, that RAC with relatively high performances should be characterised by: (i) the addition of only minor fraction of coarse size RCA and (ii) increasing cement content [30,44,45].

The replacement of VA with RA made of masonry (RMA: recycled masonry aggregates) has also been repeatedly investigated. It is determined that the typical high porosity of masonry, produced by firing clay-rich raw materials, is the most limiting factor for high-performant RAC, due to the significant increment of water absorption [33]. However, Gomes and de Brito [32] produced two types of concrete made of coarse RCA and coarse RMA, with up to a 75% substitution of VA by RMA, and they did not observe significant variations in compressive strength. For substitutions greater than 75%, RAC with RMA showed a more pronounced decrease of compressive strength compared to those made of RCA. Alves et al. [34] analysed concrete produced with mixed and coarse brick RA *versus* RA made of sanitary ware. At 28 days, the decrease of compressive strength was 10% and 42.5% in the former and the latter cases, respectively.

Bravo et al. [11] analysed the mechanical performance of concrete produced with RA from mixes of CDW from various recycling plants. They observed that mixes rich in masonry materials weaken the internal microstructure of RAC, with a decrease in density and mechanical strengths. Conversely, other studies have shown that RMA, in substitution of given amounts of VA, can also increase the mechanical performance. In line with this finding, Medina et al. [43] produced concrete with 25% coarse RMA and observed an increase of compressive strength up to 11%. Thus, it appears that the role of RMA on the preparation and performance of RACs is less established and predictable than that of RCA. Again, these apparent discrepancies can be interpreted only if types and amounts of phases in aggregates are measured. Overall, it is possible to conclude that the presence of fine RAs induces a decrease of the performance of RAC, while RACs prepared with an equal amount of RCA are generally better than those with RMA. However, this general reappraisal is still qualitative and does not consider the mineralogical and petrographic nature of RA, which can be highly variable [6] and can explain divergent mechanical features of RAC [46].

## 3. Materials and Methods

### 3.1. Aggregates

VA is a natural sand cropping around Lisbon: it was used to prepare the reference mortar (RM). The post-seismic CDW rubbles investigated in this study were instead stored at the COSMARI industrial plant sited in Tolentino (Macerata, Marche region, Northern-Central Italy). COSMARI was committed for collecting, treating and storing CDW rubbles in the areas (Italian administrative provinces) of Macerata, Fermo and Ascoli Piceno (southern sector of the Marche region). Any incoming CDW rubble was manually liberated from plastic, glass, wood, textile, asphalt and asbestos-bearing materials by the operators of COSMARI. Then, inert and ceramic-like CDW fractions were crushed to obtain cm-sized grains and were accumulated in heaps by plant (Figure S1).

Representative samples for a total of about 250 kg of these heterogeneous coarse-crushed post-seismic CDW rubbles were collected, according to their mesoscopic and qualitative appearance in the field [6]. Successively, they were manually and mesoscopically sorted in six groups (about 40/45 kg per group), enriched in the NS, CO, TI, BR, PF and RT ceramic-like materials. Here, brick and perforated bricks are considered separately, although they are both masonries, while tiles and sanitary ware are considered within the same TI group. Each CDW group was first washed to remove fines and then dried.

### 3.2. Mesoscopic Analysis of RA and Quantification of Crystalline Phases by XRPD

Each group of the six RAs was sampled before the complete crushing and storing process performed by COSMARI SRL Hence, the most representative pre-crushing decimetre-sized RA CDW clasts were sampled according to their mesoscopic appearance in the field. The global similarities and differences of CDW clasts per group are reported in Table S1. The mesoscopic textures correspond to the classical eye-observation in the field on colour(s) and possible visualization (phaneritic) or not (aphanitic) phases. The density of each of these CDW clasts is also reported in Table S1 together with the colour of the resulting powders.

The identification and quantification of crystalline phases in each group of RAs were performed by X-ray Powder Diffraction and the Rietveld method, by using a representative aliquot of about 0.5 kg. About 2 g of each initial 0.5 kg were powdered using a manual agate mortar and pestled to obtain a fine and uniform powder, with crystallites’ sizes of few μm (ideally 1–10 µm). Each fine powder was side-loaded into the 15 diameter 1.5 mm deep cavity of PMMA sample holders. The powder diffractometer used was a D8 DaVinci by Bruker AXS GmbH (Karlsruhe, Germany), equipped with the CuKα X-ray tube source, the LynxEye XE 1D array detector, with the Ni filter, in the Bragg-Brentano θ-θ configuration. Each XRPD pattern was collected from 3 to 90° of 2θ, with a step scan of 0.02° and a 0D-detector equivalent counting time of 10 s per step. The obtained XRPD patterns were first checked for non-crystalline content by carefully inspecting the background. Only some of the patterns of the tile (TI) samples exhibited a visible bump related to the X-ray amorphous content. In any case, no internal standard addition was performed to achieve the absolute quantification of glassy (and crystalline) phases in these samples. The crystalline phases were, therefore, identified by a combined automatic search-match and manual search approach using the Bruker DIFFRAC.EVA v. 6.0.0.7 software and the PDF-2 database. The QPA-Rietveld of crystalline phases (wt.%) was performed using Profex, a graphical user interface for the Rietveld refinement program, BGMN [47], according to the guidelines in [48]. An example of final Rietveld fit is reported in Figure S3.

### 3.3. Preparation of Mortars

**Figure 1:**
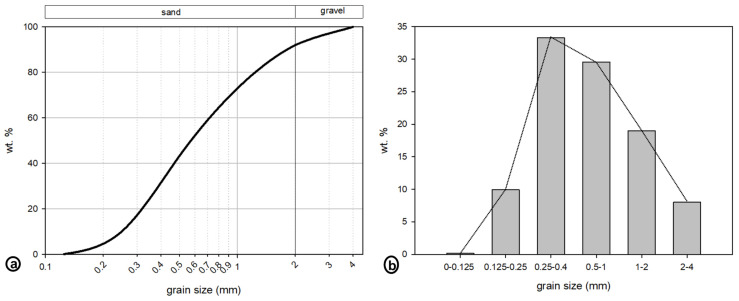
(**a**) Cumulative grain-size curve (Fuller grain-size distribution) of the aggregates used to produce recycled mortars. (**b**) Grain sizes by wt.%.

The VA, the six RA groups and the six groups made of 50 wt.% of VA and 50 wt.% of each of the CDW groups were used to prepare 13 different aggregates (Figure S2). Each group of RAs was further crushed and sorted in order to obtain a Fuller grain-size distribution (Figure 1). The same cement type (CEM I 42.5 R) was used to prepare the 13 different mortars: one RM, six RAMs and six RVAMs (Figure S2). The absolute and relative contents of aggregates, cement and water are reported in Table 1. The ratio between water and cement was determined based on the results of the consistence test, which induced ratios ranging from 0.50 up to 0.65 (Table 1). All mixes were produced with a consistence of 150 ± 25 mm, to be more fairly compared. This involved a preliminary stage in which the mixing water of each mix was adjusted, whenever necessary, to comply with this requirement. The mixes of cement, water and aggregates were used to prepare a 16 × 4 × 4 cm^3^ rectangular prism of mortar specimens. Twelve mortar specimens were prepared for each of the 13 groups, summing up to 156 mortar specimens.

### 3.4. Physico-Mechanical Tests

The measurements of the physico-mechanical properties of VA, RA, RM, RVAM and RAM, as well as the preparation procedures of the mortars, refer to international standard guidelines (Table S2). The density (kg/m^3^) and water absorption (%) of aggregates (VAs and RAs), measured with a pycnometer after immersion in water for 24 h, are reported in Table 2. The consistence of each mortar was determined simultaneously during the production of the RM, RAM and RVAM specimens. At 24 h after production, the RM, RAM and RVAM specimens were extracted from the moulds.

For each of the 13 groups, 3 specimens were placed in the dry chamber at 20 ± 2 °C and a relative humidity of 50 ± 5% for the measurement of shrinkage, using the Digital Length Comparator, with daily measurements up to 28 days and weekly from 28 to 91 days. The other 9 specimens were placed into the wet chamber at 20 ± 2 °C and a humidity of 95 ± 5% for successive physico-mechanical tests, i.e., density (kg/m^3^), compressive and flexural strengths, ultrasonic pulse velocity (UPV), electrical resistivity and dynamic modulus of elasticity (Table S3). The compressive and flexural strength tests were performed at 7 and 28 days, with the FORM + TEST prufsystem M-10 instrumentation by Seidner&Co GmbH (Riedlingen, Germany). For the determination of the compressive and flexural strengths, each RM, RAM and RVAM specimens were subjected to increasing pressure with a speed of 1 kN/s and 0.05 kN/s, respectively. The ultrasonic pulse velocity (UPV), resistivity and modulus of elasticity were measured only at 28 days. The UPV test was performed using the PUNDIT (Portable Ultrasonic Non-Destructive Digital Indicating Tester) Lab/PL-1010 instrumentation by UTEST (Ankara, Turkey), setting the waveform at a 54 kHz frequency and 25 μs time. The resistivity test was conducted with electrical resistivity readings taken with a Wenner principle-based four-point device, in conditions of natural wet surface of the mortar sample. The modulus of elasticity was obtained using the GrindoSonic MK5 instrumentation by J.W. Lemmens N.V. (Leuven, Belgium), with an excitation hammer of 50 gr. The density of each mortar sample was determined at 28 days.

## 4. Results

### 4.1. Petrographic and Mineralogical Characteristics of the RA

The mesoscopic texture and colour of the 28 CDW RA samples used for XRPD per group are reported in Table S1. RA-NS-100, RA-CO-100 and RA-TI-100 are grey coloured to light brown, or even white. By contrast, RA-BR-100, RA-PF-100 and RA-RT-100 are invariably coloured from brownish to ochre up to reddish (Table S1). TI, PF and RT are invariably aphanitic and CO and BR are always porphyric to aphanitic, with colours ranging from white to grey. The results of the QPA-Rietveld analysis of the six RA groups of samples RA-NS-100, RA-CO-100, RA-TI-100, RA-BR-100, RA-PF-100 and RA-RT-100 are displayed in Figure S4a–f and Table 3. VA is rich in quartz, feldspars and sheet-silicate (Table 3).

The crystalline phases in whitish-to-pale yellowish RA-NS-100 (Table S1) are displayed in Figure S4a, and the accurate phase fractions of QPA-Rietveld are given in Table 3. These CDW samples are also mainly composed of calcite (Figure S4a). However, the amount of calcite ranges from about 50 to 80 wt.%; the decreasing of calcite is counterbalanced by the increasing of quartz, feldspars (anorthite and albite) and sheet-silicates (mica, serpentine and chlorite) (Table 3). These crystalline phase assemblages are typical of limestone to marly-limestone rocks, which are common in the areas around the CDW plant.

The six RA-CO-100 samples are similar among themselves (Figure S4b), with a typical greyish appearance (Table S1). As expected, the most abundant crystalline phase in them is calcite, with amounts >85 wt.% (Table 3). This reflects both the composition of local natural aggregates used for concrete production and the effect of the carbonation process of cement hydration products. Quartz is the second component being abundant in siliceous sands, a common aggregate used in mortars, along with the other silicates (micas, feldspars) in minor fractions (Figure S4b; Table 3). The minor occurrence of typical cement phases (gypsum, ettringite and portlandite) can be regarded as a residual after aggregate washing due to the binder fraction attached to the clasts. If the finer aggregate sizes had not been washed away, a much larger abundance would have been expected for these cement hydration phases.

The group of RA-TI-100 was analysed via five samples, displayed in Figure S4c, while their crystalline contents are reported in Table 3. The five RTs display the most variable mineralogy, with significant variations in calcite (4 to 39 wt.%), mainly counterbalanced by quartz and cristobalite (32 to 66 wt.%). The other crystalline phases are feldspars (anorthite and orthoclase), followed by clinopyroxene and traces of melilite and micas (Table 3). The most peculiar crystalline phase in RT is the significant amount of mullite, ranging from 11 to 18 wt.% (Table 3). The presence of mullite and minor quantities of cristobalite silicate phases point out that these RAs are enriched in ceramic tiles and sanitary wares. This is also confirmed by the intensity bump observed on the XRD patterns of these materials, indicating the presence of a glassy phase (Figure S4c). However, the nature of the other occurring crystalline phases, in particular the relative high abundance of calcite, suggest that these aggregates are mixtures of different kinds of ceramic-like materials and possibly calcite-rich natural stones.

The mineralogy of RA-BR-100 was obtained through three samples and their XRPD patterns are shown in Figure S4d. Their mineralogical compositions are relatively similar, with a significant content of calcite (~12 wt.%), quartz (~22 wt.%), anorthite (~25 wt.%), clinopyroxene (~12 wt.%), melilite (~9 wt.%), mica (~16 wt.%) and minor amounts of gypsum and mullite (Table 3). These bricks were manufactured by firing similar mixtures of clays and carbonate under relatively mild thermal conditions. The significant occurrence of gypsum is related to the efflorescence product that commonly affects bricks [49]. The coloured appearance of these three CDW RAs (Table S1) reflects the presence of Fe-bearing phases, e.g., Fe-bearing melilite and pyroxene (fassaite) and undetected poorly crystalline iron oxides (e.g., hematite).

The XRD results for the four brownish and aphanitic perforated brick RA-PF-100 samples (Table S1) are plotted in Figure S4e. As predictable, the mineral phase composition is similar with BR and among themselves, i.e., a significant fraction of calcite (~12 wt.%), quartz (~23 wt.%), anorthite (~28 wt.%) and clinopyroxene (~19 wt.%), a moderate amount of melilite (~9 wt.%) and mica (~6 wt.%), plus a small content of gypsum and mullite (Figure S4e, Table 3). The major difference between BR and PF is the minor amount of the clay mineral (mica) in the latter RAs. This is likely due to the higher firing temperature that is experienced by the perforated brick walls compared to the interiors of solid bricks.

The five aphanitic and brownish RA-RT-100 samples (Table S1) contain similar types and amounts of crystalline phases among themselves, as well as with BR and PF recycled aggregates (Figure S4f, Table 3). Specifically, the RTs are richer in calcite, clinopyroxene and melilite and are more depleted in quartz, mica and gypsum than BR and PF (Table 3). Janssen et al. [49] demonstrated that both brick and mortar may provide a gypsum source, respectively, via the dissolution of anhydrite and the carbonation of ettringite [49]. In the case of roof tiles, the latter source is lacking.

### 4.2. Density and Water Absorption

The density of VA, RA-NS-100, RA-CO-100 and RA-TI-100 decreases from 2600 to ~2300 kg/m^3^, whereas that of RA-BR-100, RA-PF-100 and RA-RT-100 is equal to or even less than 1900 kg/m^3^ (Table 2). In parallel, the water absorption of aggregates at 24 h is very low for VA (~0.5 wt.%), low for NS and TI (~2 wt.%), intermediate for PF (~3.6 wt.%) and higher than 7.5 wt.% for CO, RT and BR (Table 2). The mortars’ density was measured at 28 days. The RM made of only VA has the highest value, 2260 kg/m^3^; the mortars made with NS have just slightly lower values (2240 kg/m^3^); whereas the mortars made exclusively of RA of concrete (RAM-CO) and tiles (RAM-TI) have values between 2200 kg/m^3^ and 2180 kg/m^3^, respectively (Table 4).

The other three types of RAMs have a density significantly lower than the previous ones, ranging from 2050 kg/m^3^ to 2090 kg/m^3^ (Table 4). The RAVM samples show a density intermediate between those of RM and RAM, made entirely with the corresponding RA (Table 4). The density of mortars reflects the densities of aggregates (Table 2).

### 4.3. Shrinkage

**Figure 2:**
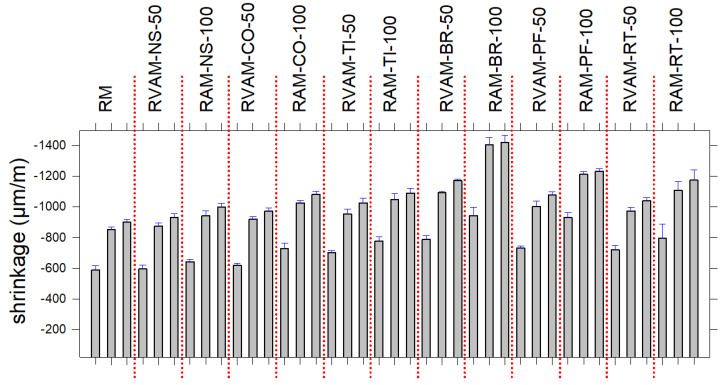
Shrinkage for RM, RVAM and RAM (Table S3) at 14 (left bar), 28 (central bar) and 91 (right bar) days.

The shrinkage values and trends at 14, 28 and 91 days are displayed in Figure 2. Overall, the shrinkage rates of all mortars are high until 28 days and then change poorly from 28 to 91 days (Figure 2, Table S3). The highest shrinkage occurs for RAM-BR-100, 1.421 µm/m at 91 days, followed by that of RAM-PF-100, 1.233 µm/m at 91 days and then RAM-RT-100, 1.175 µm/m at 91 days. Then, the RAM-TI-100 and RAM-CO-100 values are lower than the previous three RAMs and are very similar between them, being 1.090 µm/m and 1.082 µm/m at 91 days, respectively (Figure 2, Table S3). Finally, RAM-NS-100 and RM display the lowest values of shrinkage, 0.998 and 0.902 µm/m at 91 days, respectively (Figure 2, Table S3). The trends of shrinkage of mortars made of both VS and RA, i.e., RVAMs are invariably lower than that of the corresponding VA-free ones (RAMs) (Figure 2, Table S3).

### 4.4. Compressive Strength

**Figure 3:**
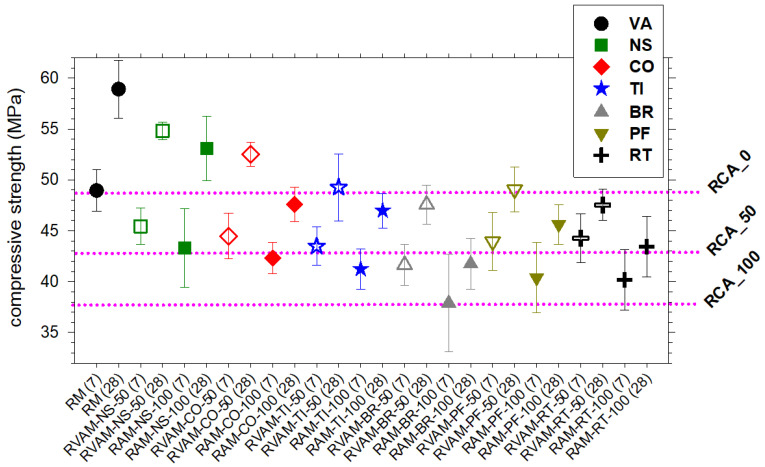
Variation of compressive strength for RM, RVAM and RMA. Unfilled symbols refer to RVAM and filled symbols refer to RAM. The horizontal pink lines correspond to the compressive strength of RACs prepared with normal strength RCAs and VA in the fraction of 0 and 100 wt.% (RCA_0), 50 and 50 wt.% of RCA and NA (RCA_50) and entirely with RCA (RCA_100), as reported in Figure 15.1 of [27].

As expected, the compressive strength at 7 days is invariably lower than at 28 days. It increases by about 10 MPa (between 20% and 23%) for RM and both NS-bearing mortar samples and more limitedly (between 7% and 17%) for all other mortars (Figure 3, Table S4). At 28 days, RM shows the highest compressive strength (~59 MPa), followed by RAM-NS-100 (~53 MPa) (Figure 3, Table S4). Then, the compressive strength decreases in the following order ~48 MPa, ~47 MPa, ~46 MPa, ~43 MPa and ~42 MPa for RAMs made of CO, TI, PF, RT and BR (Figure 3, Table S4).

The strength of all RVAMs (made of both VA and RA) is invariably higher than that of the corresponding RAM (made exclusively of RA) (Figure 3, Table S4). On the whole, the compression of mortar specimens decreases when VA are progressively replaced by NS, CO, TI, PF, RT and BR. This is due to the RA’s composition and the increase of the effective ratio w/b as the RA incorporation increases. However, the results demonstrate that the scale of the compressive strength’s decrease varies with several factors. One of the factors that most influenced the results was the RA’s source. The compressive strengths of the RAMs and RVAMs prepared here are invariably higher than those of typical RACs made of only CDW as reported in the literature, such as in [27], and are shown for comparison in Figure 3.

### 4.5. Flexural Strength

**Figure 4:**
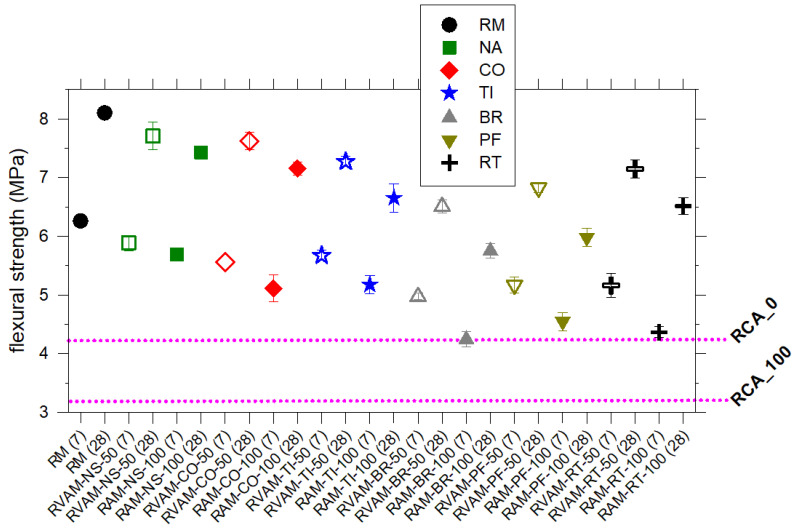
Variation of flexural strength of RM, RVAM and RAM. Unfilled symbols refer to RVAM and filled symbols refer to RAM. The horizontal pink lines correspond to the compressive strength RAC prepared with normal strength RCA and NA in the fraction of 0 and 100 wt.% (RCA_0) and entirely with RCA (RCA_100), as reported in [27].

The flexural strength of all RM, RAM and RVAM shows a similar behaviour to compressive strength (Figure 3 and Figure 4). The flexural strength invariably increases by about 1/2 MPa from 7 to 28 days (Figure 4). In line with the compression behaviour, all the RVAM are invariably 1/2 MPa higher than the corresponding RAM (Figure 4, Table S5). At 28 days, RM shows the highest flexural strength at about 8 MPa, closely followed by 7.4 MPa of RAM-NS-100 (Figure 4, Table S5); the flexural strength of RAM decreases sequentially from RAM-CO-100, RAM-TI-100, RAM-RT-100, RAM-PF-100 to RAM-BR-100, respectively, at 7.2 MPa, 6.7 MPa, 6.5 MPa, 6.0 MPa and 5.8 MPa (Figure 4, Table S5). This is due to the increase in the effective w/b of these mixes and the negative effect of the composition of some of the RAs.

The flexural strength values of the RAM and RVAM prepared here are largely and invariably higher than those of typical RACs made of only CDW as reported in the literature, e.g., [27], as well as of RAC made exclusively of VA (Figure 4).

### 4.6. Ultrasonic, Electrical Resistivity and Dynamic Modulus of Elasticity Characteristics

**Figure 5:**
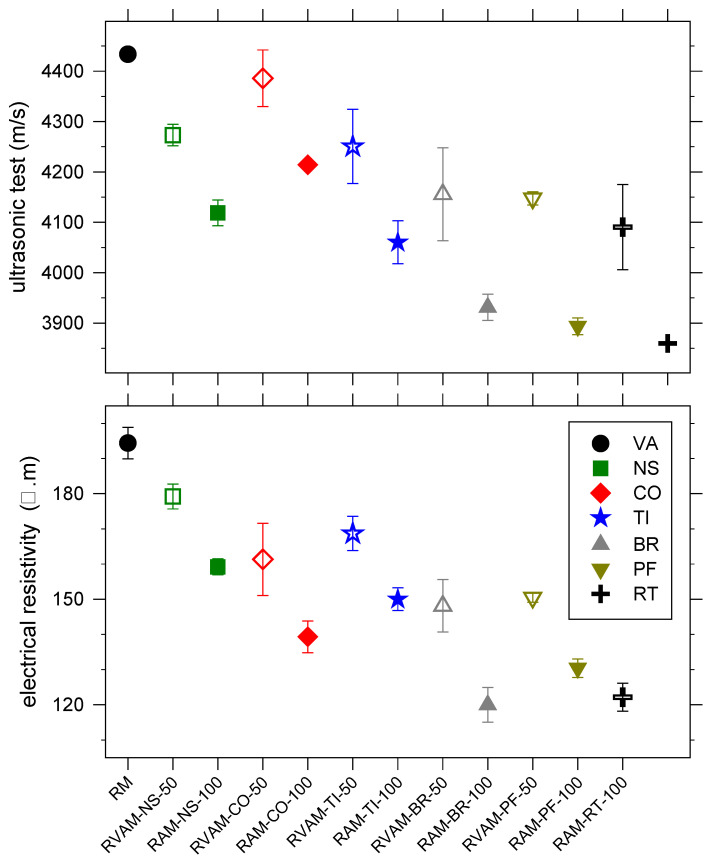
Variation of ultrasonic pulse velocity (**top**) and electrical resistivity (**bottom**) for RM, RVAMs (unfilled symbols) and RAMs (filled symbols).

**Figure 6:**
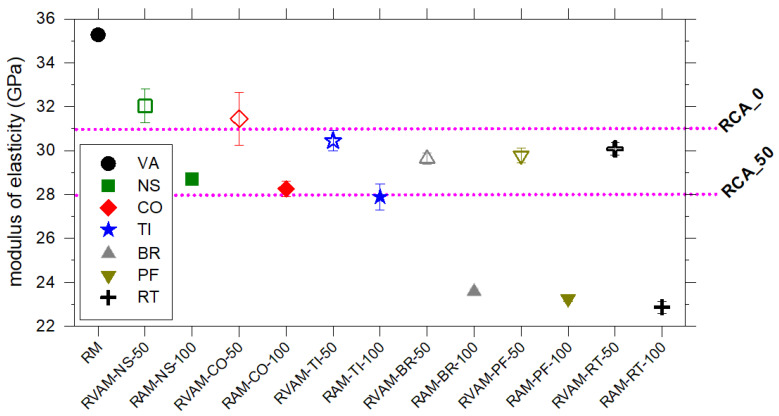
Variation of modulus of elasticity for RM, RVAM (empty symbols) and RAM (filled symbols). The horizontal pink lines correspond to the modulus of elasticity of RACs prepared with normal strength RCAs and VAs in the fraction of 0 and 100 wt.% (RCA_0) and 50 and 50 wt.% of RCA and NA (RCA_50), as reported in [27].

The UPV, electrical resistivity and dynamic modulus of elasticity were all determined at 28 days (Figure 5 and Figure 6, Tables S6 and S7). The UPV values of all RM, RVAM and RAM range between 4434 m/s and 3860 m/s; the RVAM have higher UPV values than the counterpart RAM specimens (Figure 5, Table S6). The UPV decreases sequentially from RM, RAM-CO-100, RAM-NS-100, RAM-TI-100, RAM-BR-100, RAM-PF-100 to RAM-RT-100 (Figure 5, Table S6). The UPV results are consistent with those observed for compressive strength; lower UPVs were obtained with decreasing compressive strength. As the RA content increased, the UPV decreased. This decrease is due to the greater porosity of the matrix.

The electrical resistivity ranges from 194 Ω·m for RM to 120 Ω·m for RAM-BR-100; again, the RVAMs have higher resistivity values than the counterpart RAM specimens (Figure 5, Table S6). The electrical resistivity of mortars made with only RA decreases sequentially from RAM-NS-100, RAM-TI-100, RAM-CO-100, RAM-PF-100, RAM-RT-100 to RAM-BR-100 (Figure 5, Table S6). Using RA induced a decline in resistivity due to its higher porosity.

The dynamic modulus of elasticity ranges from 35 GPa for RM to 23 GPa for RAM-RT-100; the RVAMs have a modulus of elasticity that is higher than their corresponding RAM specimens (Figure 6, Table S7). The dynamic modulus of elasticity of RAMs decreases in the following order: RAM-NS-100, RAM-CO-100, RAM-TI-100, RAM-BR-100, RAM-PF-100 and RAM-RT-100 (Figure 6, Table S7). The modulus of elasticity of mortars prepared with NS, CO and TI with and without VA show similar values with those reported in the literature made of only VA and VA-plus-RA. The same situation holds for RVAMs made with BR, PF and RT, whereas our RAMs made exclusively of BR, PF and RT have very low values (Figure 6).

## 5. Discussion

**Figure 7:**
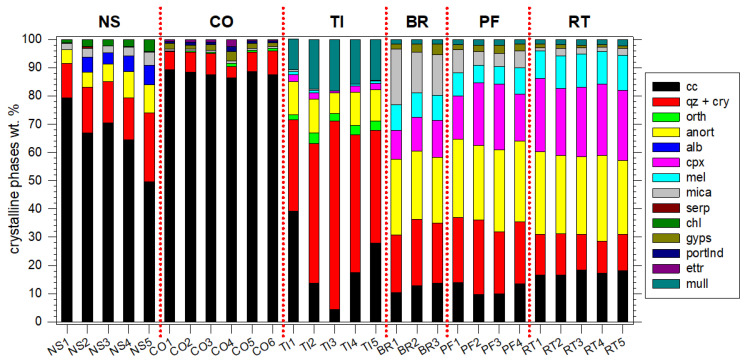
QPA-Rietveld abundance of crystalline phases in the six groups of RAs from CDW rubbles. The acronyms of crystalline phases are reported in Table 3.

A general and direct reappraisal of the amounts of the crystalline phases in the six groups of RAs, as obtained by XRPD and QPA-Rietveld, is provided in Figure 7 and resumed in Table 3. The VA sample is by far the richest in quartz, followed by feldspar phases and the trace of a sheet-silicate phase (Table 3). The most abundant and common crystalline phases in RA-NS-100 are calcite and feldspars (quartz, anorthite and albite), plus minor other phases; the abundance of calcite strongly increases for RA-CO-100 RA (Figure 7). The RA-BR-100, RA-PF-100 and RA-RT-100 show similar types and amounts of crystalline phases, i.e., calcite, feldspars, clinopyroxene and sheet-silicates (mica) (Figure 7). The RAM-RT-100s are instead different from all the other five CDWs, since they are the richest in feldspars, mullite and glass (the latter not quantified), plus calcite and other minor crystalline components (Figure 7).

The relatively low content of calcite in these four types of construction materials mirrors the bulk compositional features of their source materials [4,6,14,50]. Clays and claystones are by far the most abundant raw materials used to fabricate bricks, perforated bricks, roof tiles and tiles. In clay-rich deposits, the content of carbonates is commonly limited due to the high temperatures’ firing of clay-rich raw materials which induce a decomposition of carbonates [12,13,51,52]. In turn, the presence of calcite reflects the presence of NS and CO in CDW enriched in BR, PF, RT and, especially, TI. The remarkable differences in the crystalline and non-crystalline phase composition between the six groups of RAs considered here are clearly originating from the different sources of the raw materials and the different industrial production processes of building components [53]. Hence, the silicate and carbonate crystalline phases are those already present in raw materials (natural stones and aggregates), formed during hardening of cements (mortars and concrete) and/or produced or lost (dehydration and decarbonation) at high temperatures during their preparation (bricks, roof tiles and tiles).

Mullite and melilite, plus parts of SiO_2_ polymorph, feldspars and clinopyroxenes detected and quantified in Figure 7, commonly result from high-temperature industrial treatments, whereas calcite and part of quartz, feldspars and sheet-silicates (mica) are those already present in raw materials [51]. The different relative abundances of mineral phases also explain the large chemical variations within the CDW rubbles. Specimens enriched in calcite are also CaO and CO_2_-rich. The abundance of calcite in RA-NS-100 and RA-CO-100 sampled at COSMARI reflects the widespread occurrence of limestones in this geographical area of provenance [6], e.g., northern Abruzzo and southern Marche plus Umbria regions (Italy). These regions are characterised by extensive outcropping of carbonate rocks, i.e., limestones, used and excavated for a long time to produce aggregates for concrete [54], as well as ornamental or block stones for buildings. The high content of calcite, or in general of carbonate crystalline phases, commonly Fe-free, determines the whitish to greyish appearance of these construction materials (Table S1).

Therefore, the coupling between mesoscopic colours and mineralogical features allows concluding that most CDW earthquake-derived rubbles composed of natural stones and concrete from this region are mainly colourless and rich in carbonates and/or Fe-free (feldspars) or -poor (sheet- and chain-silicates) phases (Figures S1 and S2). On the other hand, the colour of RA-TI-100 samples spans from white to grey to brown (Table S1), reflecting the presence of a minor Fe-bearing crystalline phase (Table 3, Figure S2 and Figure 7). The deep reddish to brownish colours of BR, PF and RT are instead linked to the presence of significant amounts of Fe-bearing minerals, such as clinopyroxene, mica and possibly melilite (Table S1 and Table 3, Figure S2 and Figure 7). In addition to the same light or colourless tints, RA-NS-100, RA-CO-100 and RA-TI-100 also have a similar density; by contrast, the density of coloured BR, PF and RT as well as RA are both low (Table 2 and Table 4), respectively. These differences in colour and density of RA-NS-100, RA-CO-100 and RA-TI-100 *versus* RA-BR-100, RA-PF-100 and RA-RT-100 are key physical attributes for their possible optical/density sorting in industrial plants. Moreover, the separation of NS and CO, such as those investigated here, can be further refined from masonry using chemical attributes via hyperspectral analysis, since the former are mainly CaO-rich [55,56,57,58,59,60].

As reported or summarised in previous studies [7,33,35,36,61,62] and confirmed by the present work, RAM prepared with BR, PF and RT displays compressive and flexural strength values lower than mortars with NA, CO and TI (Figure 3 and Figure 4). In a similar way, the other physical and mechanical parameters follow similar behaviours (Figure 2, Figure 5 and Figure 6). Commonly, RAs show a water absorption that is significantly higher than VAs [7,61,62,63]. This can be attributed to the high porosity of CDW due to the presence of attached mortars; nonetheless, the higher porosity and water absorption of CDW can also be due to petrographic features of these materials [12,13,14,50,52]. The porosity of CDWs and their ability to absorb a high quantity of water are invoked to induce low mechanical performances of RACs or RAMs. In general, the higher the porosity of a CDW is, the lower the mechanical performance of a RAC is [8,26,62,63,64,65,66]. Our findings confirm this general relationship, but it can be further clarified and expanded here.

The water absorption of RA-NS-100 and RA-TI-100 is the lowest among RAs, followed by RA-PF-100, RA-CO-100, RA-RT-100 and RA-BR-100 (Table 2). Since the water absorption of calcite grains is very limited, the water absorption of the RA-CO-100 group is entirely attributable to the attached mortar, which is consistent with the detection of cement hydration phases by XRPD (Table 3, Figure S4b). The mortar specimens prepared with RA-CO-100 have significantly higher compressive and flexural strengths, in spite of the high water absorption (7.4%), like those of RA-BR-100 and RA-RT-100 (Table 2 and Table 4, Figure 3 and Figure 4). Such an unexpected and unprecedented outcome is attainable only by mineralogical determinations.

This information straightforwardly indicates that porosity and water absorption affect physico-mechanical characteristics of RAM and RAC [26,33,62,63,64,65,66], but other attributes also play a crucial role. Similarly, the VA and RA-NS-100 have very similar density and water absorption. However, their differences in compressive and flexural strengths are small but detectable (Figure 3 and Figure 4). These variations can be only attributed to the differences in the mineral composition of the aggregates. In line with this, VA and RA-NS-100 are mainly made by quartz and calcite, respectively (Table 3), plus some attached mortar in NS, responsible for water absorption of 1.5% (Table 2).

The differences in the crystalline phases of these various types of aggregates determine differences in the texture and phases developed in the interfacial transition zone (ITZ) between aggregates and cement paste, during the setting (hardening) of mortars [61,62,67]. The formation of calcium hydroxide and silicates in the ITZ between aggregate and cement paste allows obtaining good physico-mechanical properties of the mortar/concrete [61,62,67]. The XRPD analysis of the tiles shows a high content of quartz and mullite, with amorphous phases (Figure 7, Table 3). The mineralogical characteristics of tiles (RA-TI-100) could generate pozzolanic activity, able to promote significant physico-mechanical properties [5,10,61,62]. This holds for the compressive and flexural strengths displayed by tile-bearing mortars (Figure 3 and Figure 4).

**Figure 8:**
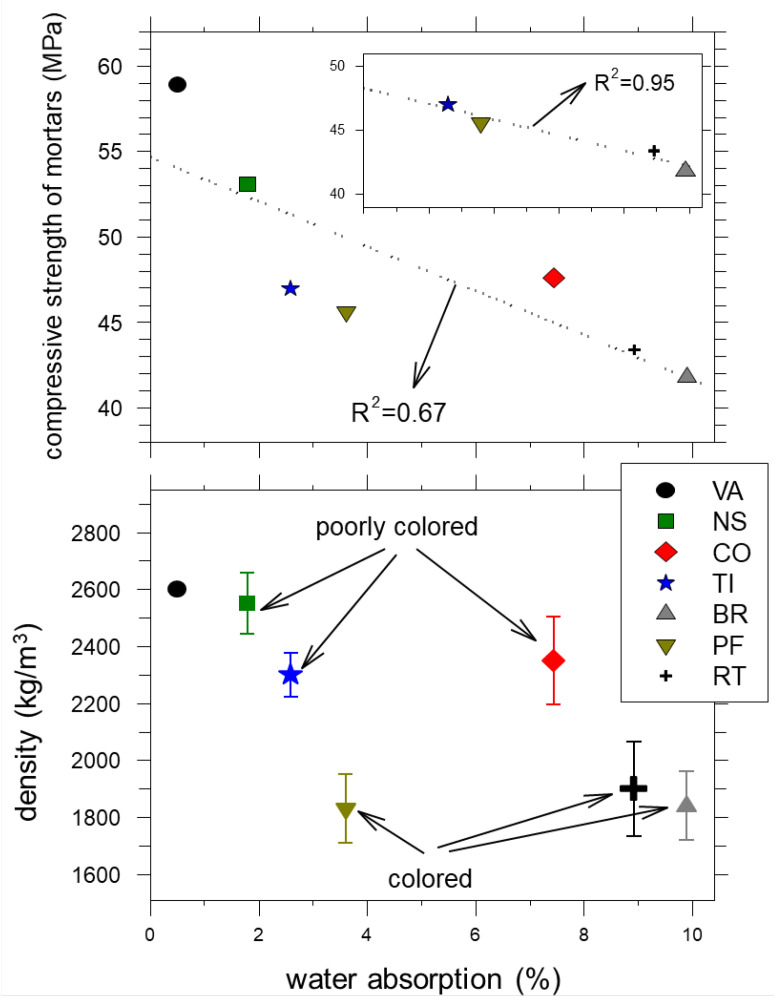
General reappraisal of mesoscopic appearance (colour), density and mass volume (bottom) and compressive strength as a function of water absorption of the six RAs.

The classical relationships between mechanical performance (compression), density and water absorption for mortars can be globally summarized here but can be rationally interpreted only in light of the petrography and mineralogy of aggregates, as shown in Figure 8. The water absorption of the six CDW groups is plotted *versus* their density (different CDW specimens), as well as their colour. RA-NS-100, RA-CO-100 and RA-TI-100 are generally lightly coloured and with a high density, whereas RA-BR-100, RA-PF-100 and RA-RT-100 are low-density and darker coloured. These attributes combine with the mechanical properties of corresponding RAMs, since the former CDW types have higher compressive strength than the latter ones (Figure 8). In general, a high water absorption determines a progressive decrease in compressive strength. However, it is noteworthy that the compressive strength of CDWs made of silicate phases and are calcite-poor or calcite-free (RA-TI-100, RA-BR-100, RA-PF-100 and RA-RT-100) fit a unique linear regression (Figure 8). On the other hand, the calcite-rich RA-CO-100, although having a high porosity, has a compressive strength even larger than silicate-rich RA-TI-100 (Figure 8).

The lower water absorption of tiles with respect to bricks and roof tiles is related to their preparation since they are fired at different conditions. Tiles are normally heated up to 1300 °C, while bricks and roof tiles are heated up to ~800 °C [10,61,62]. Again, all these construction materials are produced from clays, but the different heat treatments determine a different mineralogy and volume of pores [10,61,62]. The mineral composition shows that tiles are mainly composed of SiO_2_ plus amorphous phases (Figure 7), which allows them to acquire pozzolanic activity [5,10,61,62]. The water absorption of tiles usually shows values of ~1–2% [7,37]. In line with this, the physico-mechanical properties of RAC produced with 10 wt.% of RA deriving from tiles show no significant differences, compared to RACs produced with NA [38].

## 6. Conclusions and Outlooks

CDWs collected from earthquake rubbles are extremely heterogeneous, but if adequately characterized and separated can be used to design and produce mortars with relatively high and potentially constant performance (Figure 3 and Figure 4). The performance of RAMs can be in any case tuned and enhanced, if required, by adding virgin aggregates (RVAMs). Porosity and water absorption of a type of CDW are important [68] but not sufficient to assess the quantitative performance of an RA used to prepare new mortars (RAMs). The first ever reported quantitative phase analysis of aggregates performed by the XRPD and the Rietveld method expands these previous findings and unveils new features and relationships. Definitely, the mechanical performance of mortars prepared with VA and RA is significantly affected by their relative amounts, but also by the types and amount of phases composing them (Figure 8).

Differences in mineralogy, petrography and porosity of RA determine a significant change in the mechanical properties of RAMs. RAs rich in NS, CO and/or TI are by far more suitable to prepare new RAMs and RVAMs, whereas those rich in BR, PF and RT are much less adequate. The compressive and textural strengths and modulus of elasticity of any of these types of mortars containing CDW are comparable or even higher than those of other RACs, prepared with unsorted RCAs whose mineralogy and petrography are unknown [27]. The heterogeneity of CDW can further induce reduction of mechanical performance by the presence of localised enrichment in weak phases in derived RAMS or RACs.

In future studies, the determination of the phase composition by XRPD and QPA-Rietveld of RA will be an extremely important tool to consider and will also be valuable to discriminate and potentially sort different types of ceramic-like materials within the CDW. The knowledge of the mineralogical content is mandatory to evaluate possible reuses in new mortars or concrete. This is also of paramount importance to evaluate (or discard) the possibility of sorting different CDWs and estimating the related costs in any given geographical and geological context [9,61].

Finally, in geographical and geological areas where carbonate rocks are abundant, it is potentially possible to separate at the industrial scale, automatically and cheaply, natural stones and concrete, but possibly also tiles, from bricks, perforated bricks and roof tiles. These possibilities of advanced sorting are crucial for CDW recycling in new RACs or RAMs. The (automated) sorting of CDW must be based not only on grain-size distribution but also on other measurable attributes such as colour, density and, especially, mineralogy and petrography. The automated sorting of CDW in different material types with constant and known properties would open a unique opportunity for improving their recycling, as well as upcycling in new construction materials, and it would contribute to the economic resilience of districts, e.g., hit by earthquakes, as well as in the normal recycling of waste from the construction sector under common conditions.

## Figures and Tables

**Table 1:** Recycled (RA) and virgin (VA) aggregates, cement and water amounts used for the preparation of mortars.

Label	Type of Aggregate	RA (%)	Cement (kg)	Coarse (>0.5–≤4 mm) VA (kg)	Fine (≤0.5 mm) VA (kg)	CDW Aggregate (kg)	Water (L)	Water/Cement Ratio
RM	VA	0	1.162	1.218	1.218	-	0.581	0.50
RVAM-NS-50	NS	50	1.162	0.609	0.609	1.141	0.581	0.50
RAM-NS-100		100	1.162	-	-	2.282	0.616	0.53
RVAM-CO-50	CO	50	1.162	0.609	0.609	1.145	0.581	0.50
RAM-CO-100		100	1.162	-	-	2.290	0.616	0.53
RVAM-TI-50	TI	50	1.162	0.609	0.609	1.085	0.581	0.50
RAM-TI-100		100	1.162	-	-	2.171	0.616	0.53
RVAM-BR-50	BR	50	1.162	0.609	0.609	0.976	0.662	0.57
RAM-BR-100		100	1.162	-	-	1.952	0.732	0.63
RVAM-PF-50	PF	50	1.162	0.609	0.609	0.997	0.674	0.58
RAM-PF-100		100	1.162	-	-	1.994	0.755	0.65
RVAM-RT-50	RT	50	1.162	0.609	0.609	1.014	0.674	0.58
RAM-RT-100		100	1.162	-	-	2.028	0.755	0.65

**Table 2:** Physical properties of cement, VA and RA.

Label	Type of Aggregate	Water Absorption (%)	Density (kg/m^3^)
cement	-	-	3100
VA	natural sand of Lisbon	0.50	2600
RA-NS-100	CDW natural/ornamental stone	1.79	2550 (108)
RA-CO-100	CDW concrete	7.44	2350 (154)
RA-TI-100	CDW tile	2.59	2300 (76)
RA-BR-100	CDW brick	9.89	1840 (120)
RA-PF-100	CDW perforated brick	3.61	1830 (121)
RA-RT-100	CDW roof tile	8.92	1900 (166)

Note: the reported density is the average (variance) of measurements made on 6, 5, 5, 3, 4 and 5 different samples for RA-CO-100, RA-NS-100, RA-TI-100, RA-BR-100, RA-PF-100 and RA-RT-100, respectively.

**Table 3:** Crystalline phases and their quantitative abundance (wt.%) by XRPD data.

CDW Type	Label	cc	Qz + cri	orth	anort	alb	cpx	mel	mica	serp	chl	gyps	port	ettr	mul
VA	coarse	-	94.3 (3)	5.7 (3)	-	-	-	-	-	-	-	-	-	-	-
	fine	-	82.4 (8)	11.0 (5)	-	5.3 (6)	-	-	1.2 (3)	-	-	-	-	-	-
	average	-	88.4	8.4	-	2.7	-	-	0.6	-	-	-	-	-	-
	median	-	88.4	8.4	-	2.7	-	-	0.6	-	-	-	-	-	-
	st dev	-	8.4	3.7	-	3.7	-	-	0.8	-	-	-	-	-	-
RA-NS-100	NS1	79.4 (3)	12.2 (2)	-	4.8 (2)	0.0 (0)	-	-	2.1 (1)	0.2 (1)	1.3 (1)	-	-	-	-
	NS2	67.0 (4)	16.1 (2)	-	5.2 (3)	5.5 (3)	-	-	3.1 (1)	0.5 (1)	2.6 (2)	-	-	-	-
	NS3	70.5 (3)	14.6 (2)	-	6.1 (3)	4.0 (2)	-	-	2.5 (1)	0.0 (0)	2.3 (1)	-	-	-	-
	NS4	64.5 (4)	14.9 (3)	-	9.3 (4)	5.5 (3)	-	-	3.1 (1)	0.1 (0)	2.6 (2)	-	-	-	-
	NS5	49.5 (5)	24.6 (4)	-	9.9 (4)	6.9 (4)	-	-	4.6 (2)	0.2 (1)	4.3 (2)	-	-	-	-
	average	66.2	16.5	-	7.0	4.4	-	-	3.1	0.2	2.6	-	-	-	-
	median	66.7	15.5	-	6.6	4.9	-	-	3.1	0.2	2.6	-	-	-	-
	st dev	10.9	4.8	-	2.4	2.7	-	-	0.9	0.2	1.1	-	-	-	-
RA-CO-100	CO1	89.2 (2)	6.5 (2)	0.2 (1)	-	-	-	-	0.7 (1)	-	-	1.9 (1)	0.8 (1)	0.7 (1)	-
	CO2	88.3 (2)	7.2 (2)	0.4 (4)	-	-	-	-	0.7 (7)	-	-	1.4 (1)	1.0 (1)	1.0 (1)	-
	CO3	87.4 (2)	7.7 (2)	0.4 (1)	-	-	-	-	0.6 (1)	-	-	2.0 (1)	1.0 (1)	0.9 (1)	-
	CO4	86.3 (3)	4.0 (3)	1.3 (2)	-	-	-	-	0.9 (1)	-	-	3.3 (1)	1.6 (1)	2.6 (2)	-
	CO5	88.7 (2)	6.9 (2)	0.6 (1)	-	-	-	-	0.7 (1)	-	-	1.7 (1)	0.9 (0)	0.5 (1)	-
	CO6	87.6 (2)	8.4 (2)	0.7 (1)	-	-	-	-	0.7 (1)	-	-	1.3 (1)	0.7 (0)	0.6 (1)	-
	average	87.9	6.8	0.6	-	-	-	-	0.7	-	-	1.9	1.0	1.1	-
	median	88.0	7.1	0.5	-	-	-	-	0.7	-	-	1.8	0.9	0.8	-
	st dev	1.1	1.5	0.4	-	-	-	-	0.1	-	-	0.7	0.3	0.8	-
RA-TI-100	TI1	39.3 (3)	32.3 (3)	1.7 (2)	11.7 (3)	-	2.6 (2)	1.1 (1)	0.5 (1)	-	-	-	-	-	10.9 (4)
	TI2	13.6 (2)	49.5 (4)	3.7 (2)	12.0 (3)	-	2.2 (2)	1.0 (2)	0.3 (1)	-	-	-	-	-	17.7 (4)
	TI3	4.5 (2)	66.5 (5)	2.8 (2)	7.3 (3)	-	0.7 (2)	0.0 (0)	0.0 (0)	-	-	-	-	-	18.2 (5)
	TI4	17.5 (2)	48.8 (3)	3.2 (2)	11.8 (3)	-	2.2 (2)	0.6 (1)	0.1 (0)	-	-	-	-	-	15.8 (4)
	TI5	27.8 (3)	39.9 (4)	3.5 (2)	10.9 (3)		2.2 (2)	0.9 (2)	0.4 (1)	-	-	-	-	-	14.4 (4)
	average	20.5	47.4	3.0	10.7	-	2.0 (2)	0.7	0.2	-	-	-	-	-	15.4
	median	17.5	48.8	3.2	11.7	-	2.2 (2)	0.9	0.3	-	-	-	-	-	15.8
	st dev	13.4	12.7	0.8	2.0	-	0.7	0.4	0.2	-	-	-	-	-	2.9
RA-BR-100	BR1	10.3 (3)	20.5 (4)	-	26.7 (4)	-	10.2 (4)	9.2 (2)	19.7 (7)	-	-	1.8 (1)	-	-	1.6 (1)
	BR2	12.9 (3)	23.4 (4)	-	24.1 (4)	-	12.0 (5)	8.6 (2)	14.4 (6)	-	-	2.9 (1)	-	-	1.7 (1)
	BR3	13.6 (2)	21.4 (3)	-	23.3 (4)	-	13.0 (4)	8.8 (2)	14.6 (6)	-	-	3.7 (1)	-	-	1.6 (1)
	average	12.3	21.8	-	24.7	-	11.7	8.9	16.2	-	-	2.8	-	-	1.6
	median	12.9	21.4	-	24.1	-	12.0	8.8	14.6	-	-	2.9	-	-	1.6
	st dev	1.7	1.5	-	1.9	-	1.4	0.3	3.0	-	-	1.0	-	-	0.1
RA-PF-100	PF1	13.9 (3)	23.1 (4)	-	27.6 (4)	-	15.4 (3)	8.1 (3)	8.2 (5)	-	-	1.9 (1)	-	-	1.8 (2)
	PF2	9.8 (2)	26.3 (3)	-	26.3 (3)	-	22.2 (3)	6.2 (2)	5.0 (2)	-	-	2.1 (1)	-	-	2.1 (2)
	PF3	10.0 (2)	21.8 (3)	-	29.0 (4)	-	23.3 (3)	6.3 (2)	4.7 (2)	-	-	2.8 (1)	-	-	2.1 (2)
	PF4	13.4 (3)	22.1 (4)	-	28.6 (4)	-	16.6 (3)	9.3 (3)	6.0 (2)	-	-	2.4 (1)	-	-	1.7 (2)
	average	11.8	22.3	-	27.9	-	19.4	7.5	5.9	-	-	2.3	-	-	1.9
	median	11.7	22.6	-	28.2	-	19.4	7.2	5.5	-	-	2.2	-	-	1.9
	st dev	2.2	2.0	-	1.2	-	4.0	1.5	1.6	-	-	0.4	-	-	0.2
RA-RT-100	RT1	16.6 (2)	14.5 (3)	-	29.2 (4)	-	25.8 (3)	9.9 (2)	1.1 (2)	-	-	1.2 (1)	-	-	1.7 (1)
	RT2	16.6 (2)	14.6 (2)	-	27.8 (4)	-	23.7 (3)	11.4 (2)	2.6 (2)	-	-	1.5 (1)	-	-	1.8 (1)
	RT3	18.3 (2)	12.6 (2)	-	27.5 (4)	-	24.6 (3)	11.8 (2)	2.2 (2)	-	-	1.0 (1)	-	-	2.0 (1)
	RT4	17.2 (2)	11.4 (3)	-	30.4 (4)	-	25.1 (3)	11.7 (2)	1.4 (2)	-	-	1.0 (1)	-	-	1.8 (1)
	RT5	18.1 (2)	12.9 (2)		26.1 (3)		24.9 (3)	12.3 (2)	2.4 (2)			1.1 (1)			2.2 (1)
	average	17.4	13.2	-	28.2	-	24.8	11.4	1.9	-	-	1.2	-	-	1.9
	median	17.2	12.9	-	27.8	-	24.9	11.7	2.2	-	-	1.1	-	-	1.8
	st dev	0.8	1.3	-	1.6	-	0.8	0.9	0.6	-	-	0.2	-	-	0.2

Notes: Acronyms and ideal crystal-chemical formulas of crystalline-phases: cc, calcite CaCO_3_; qz + cri, quartz and cristobalite SiO_2_; orth, orthoclase (Na, K)AlSi_3_O_8_; anort, anorthite, CaAl_2_Si_2_O_8_; alb, albite NaAlSi_3_O_8_; cpx, clinopyroxene (Na, Ca, Mg, Fe^2+^)(Mg, Fe^2+^Al, Fe^3+^)(Al, Si)_2_O_6_; mel, melilite Ca_2_(Mg, Al, Fe) (Al, Si)O_7_; mica K(Mg, Fe^2+^,Al, Fe^3+^)_2_(Si, Al)_4_O_10_ (OH)_2_(H_2_O); serp, serpentine (Mg, Fe, Al)_2-3_(Si, Al, Fe)_2_O_5_(OH)_4_; chl, chlorite (Mg, Fe)_3_(Si, Al) _4_O _10_(OH) _2_·(Mg, Fe) _3_(OH)_6_; gyps, gypsum CaSO_4_(H_2_O)_2_; port, portlandite Ca(OH)_2_; ettr, ettringite Ca_6_Al_2_(SO_4_)_3_(OH)(H_2_O)_26_; mul, mullite (Al_4+2x_Si_2-2x_O_10-x_).

**Table 4:** Physical properties of mortars.

Label	Weight of Specimen (kg)	Density (kg/m^3^)	∆_A0_
RM	0.578	2260	−
RVAM-NS-50	0.574	2240	−1%
RAM-NS-100	0.573	2240	−1%
RVAM-CO-50	0.57	2230	−1%
RAM-CO-100	0.562	2200	−3%
RVAM-TI-50	0.565	2210	−2%
RAM-TI-100	0.558	2180	−3%
RVAM-BR-50	0.567	2220	−2%
RAM-BR-100	0.535	2090	−7%
RVAM-PF-50	0.556	2170	−4%
RAM-PF-100	0.524	2050	−9%
RVAM-RT-50	0.554	2160	−4%
RAM-RT-100	0.528	2060	−9%

Notes: The weight and density are obtained averaging three weighting measurements; ∆_A0_—indicates the variation referred to RM.

## Data Availability

Not applicable.

## Supplementary Materials

The following supporting information can be downloaded at: https://www.mdpi.com/article/10.3390/ma16072855/s1, Figure S1: On the left, a pile of post-earthquake rubble at the COSMARI public plant (Tolentino, Marche, Italy) after first processing by hand picking removal of metals, woods, plastic, etc. On the right, the CDW after crushing and size-sorting showing the extreme heterogeneity of type-unsorted ceramic-like material. Figure S2: Scheme of the 13 different types of mortars prepared with VA and the six RA. Figure S3: Example of Rietveld fit for sample RT2 (upper ticks mark the reflection lines of the quantified crystalline phases from top to bottom: gehlenite, muscovite, quartz, calcite, plagioclase, augite, mullite, gypsum). Figure S4: (a): Stacked XRPD patterns from 3 to 55 of 2θ° (the actual scans are from 2 to 90 of 2θ°) for the five RA-NS-100 samples corresponding to CDW RA sorted as natural stones (Table 2 and Table 3). The vertical lines correspond to the most intense Bragg reflections of crystalline standards, cps indicates counts per second and acronyms of crystalline phases as reported in Table 3, except feld (feldspars) that indicates either anorthite, albite and/or orthoclase. (b): Stacked XRPD patterns from 3 to 55 of 2θ° (the actual scans are from 2 to 90 of 2θ°) for the six RA-CO-100 samples corresponding to CDW RA sorted as concrete and mortars (Table 2 and Table 3). The vertical lines correspond to the most intense Bragg reflections of crystalline standards, cps indicates counts per second and acronyms of crystalline phases as reported in Table 3. (c): Stacked XRPD patterns from 3 to 55 of 2θ° (the actual scans are from 2 to 90 of 2θ°) for the five RA-TI-100 samples corresponding to CDW RA sorted as tiles (Table 2 and Table 3). The vertical lines correspond to the most intense Bragg reflections of crystalline standards, cps indicates counts per second and acronyms of crystalline phases as reported in Table 3, except feld (feldspars) that indicates either anort, alb and/or orth. (d): Stacked XRPD patterns from 3 to 55 of 2θ° (the actual scans are from 2 to 90 of 2θ°) for the three RA-BR-100 samples corresponding to CDW RA sorted as bricks (Table 2 and Table 3). The vertical lines correspond to the most intense Bragg reflections of crystalline standards, cps indicates counts per second and acronyms of crystalline phases as reported in Table 3, except feld (feldspars) that indicates either anort, alb and/or orth. (e): Stacked XRPD patterns from 3 to 55 of 2θ° (the actual scans are from 2 to 90 of 2θ°) for the three RA-PF-100 samples corresponding to CDW RA sorted as perforated bricks (Table 2 and Table 3). The vertical lines correspond to the most intense Bragg reflections of crystalline standards, cps indicates counts per second and acronyms of crystalline phases as reported in Table 3, except feld (feldspars) that indicates either anort, alb and/or orth. (f): to be deposited as Supplementary Material). Stacked XRPD patterns from 3 to 55 of 2θ° (the actual scans are from 2 to 90 of 2θ°) for the three RA-RT-100 samples corresponding to CDW RA sorted as roof tiles (Table 2 and Table 3). The vertical lines correspond to the most intense Bragg reflections of crystalline standards, cps indicates counts per second and acronyms of crystalline phases as reported in Table 3, except feld (feldspars) that indicates either anort, alb and/or orth. Table S1: CDW samples investigated by XRPD plus their mesoscopic features. Table S2: Standard guidelines and facilities for the determination of physical and mechanical properties of aggregates and mortars. Table S3: Shrinkage as a function of time for mortars. Table S4: Compressive strength as a function of time for mortars. Table S5: Flexural strength as a function of time for mortars. Table S6: Ultrasonic pulse velocity test and electrical resistivity of mortars at 28 days, Table S7: Dynamic modulus of elasticity of mortars at 28 days.
