# Supplementary material for: Physico-Mechanical Performances of Mortars Prepared with Sorted Earthquake Rubble: The Role of CDW Type and Contained Crystalline Phases
Source: Materials (Basel). 2023 Apr 3;16(7):2855. doi: 10.3390/ma16072855 (PMC10096024; doi:10.3390/ma16072855)
Supplement: Supplementary file 1 [file materials-16-02855-s001.zip › Supplementary figures.pdf]

Supplementary figures

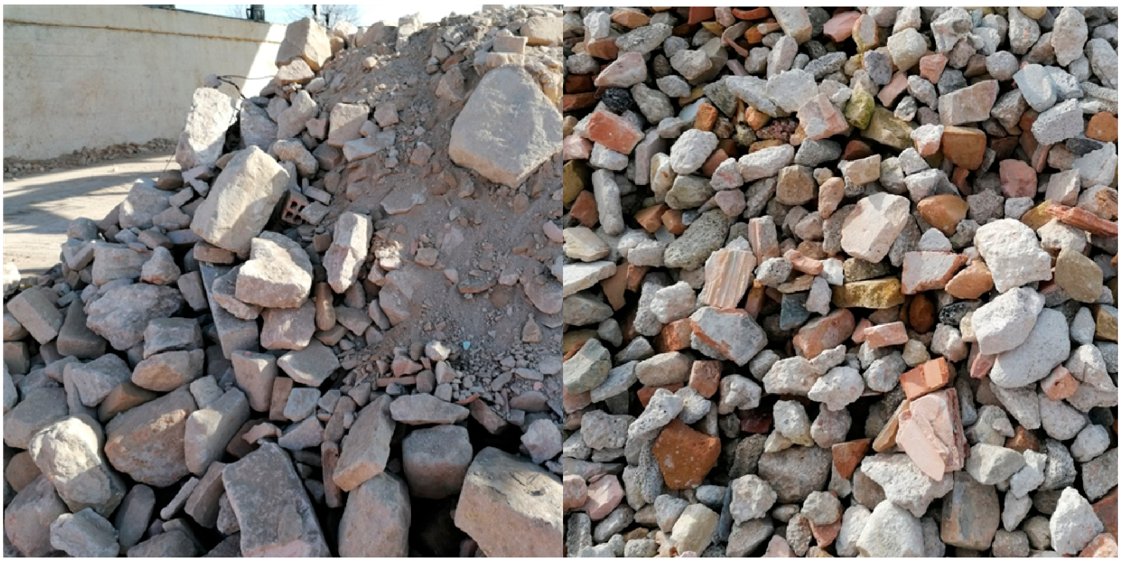

Figure S1 (to be deposited as supplementary material). On the left, a pile of post-earthquake rubble at the COSMARI public plant (Tolentino, Marche, Italy) after first processing by hand picking removal of metals, woods, plastic, etc. On the right, the CDW after crushing and size-sorting showing the extreme heterogeneity of type-unsorted ceramic-like material.

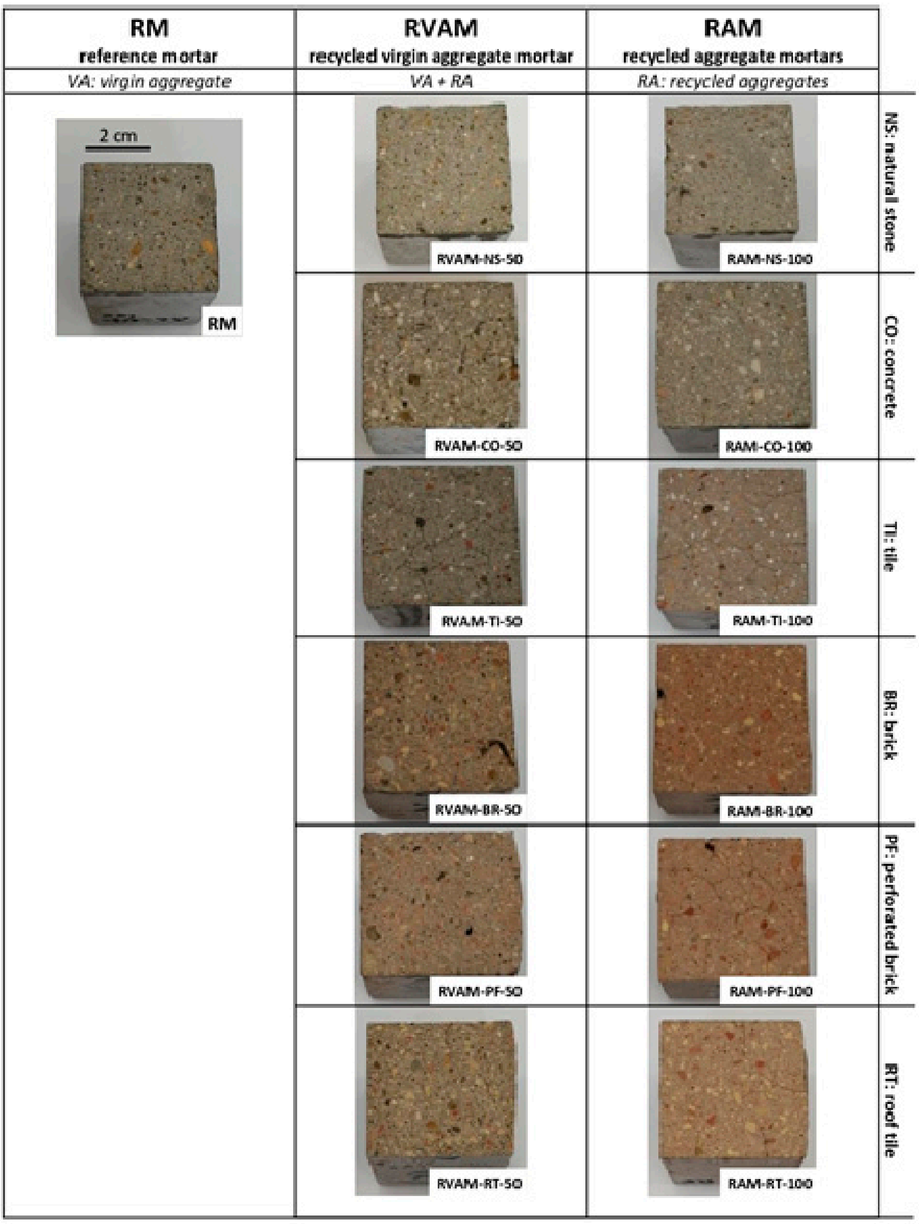


Figure S2 (to be deposited as supplementary material). Scheme of the 13 different types of mortars prepared with VA and the six RA.

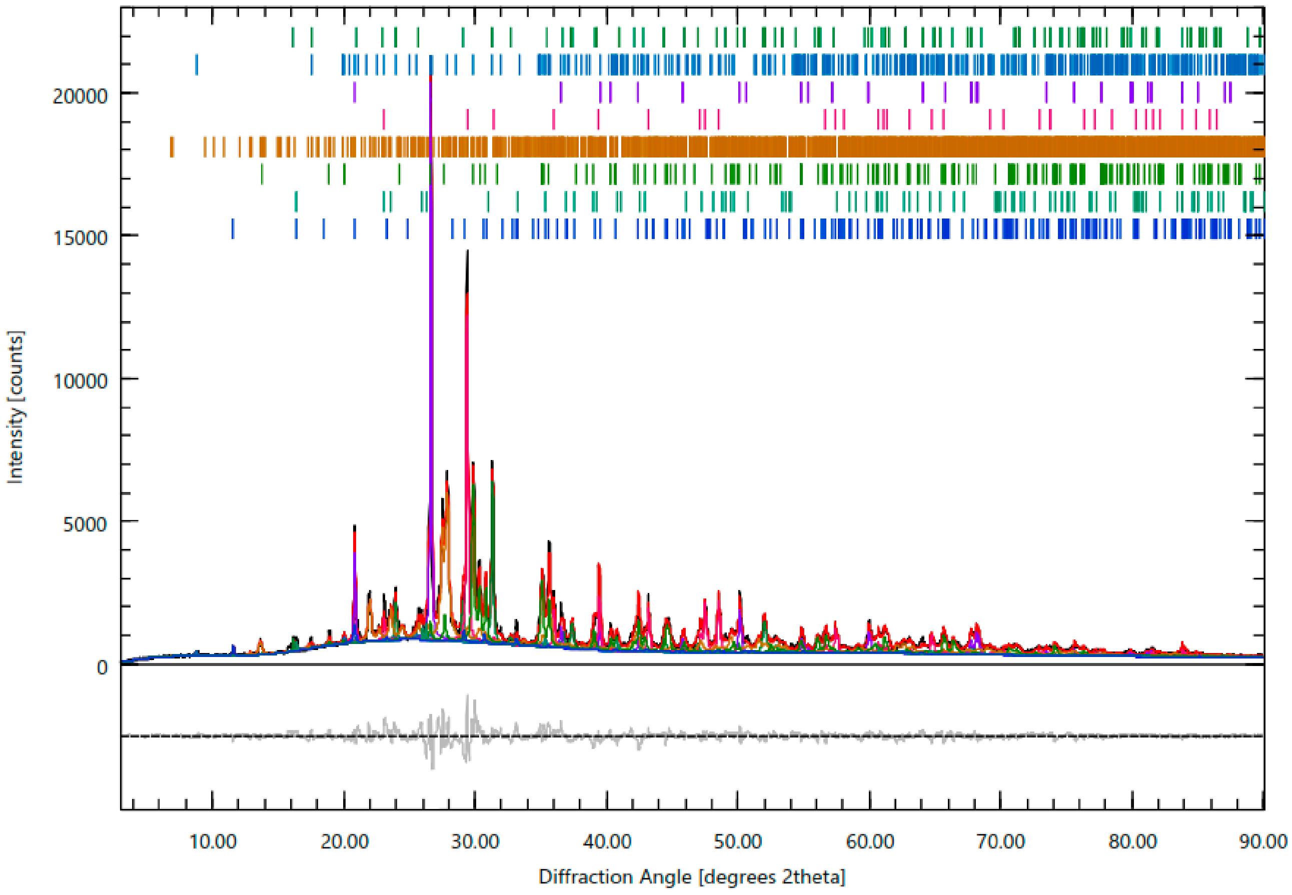


Figure S3. (to be deposited as supplementary material). Example of Rietveld fit for sample RT2 (upper ticks mark the reflection lines of the quantified crystalline phases from top to bottom: gehlenite, muscovite, quartz, calcite, plagioclase, augite, mullite, gypsum)

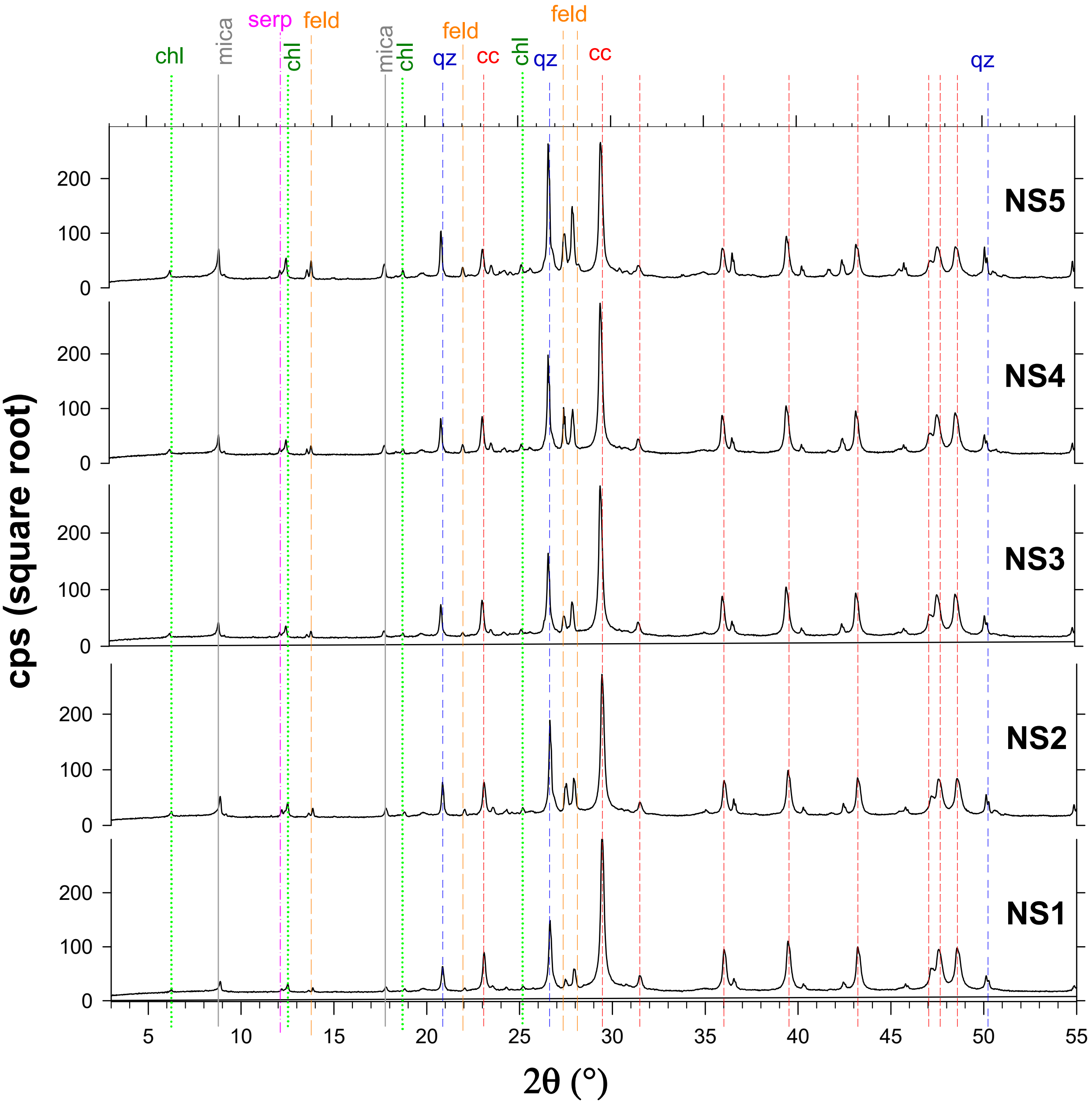

chl
mica
serp
chl
feld
mica
chl
qz
feld
cc
chl
qz
feld
cc
qz
NS5
NS4
NS3
NS2
NS1
cps (square root)
2θ (°)


(a)

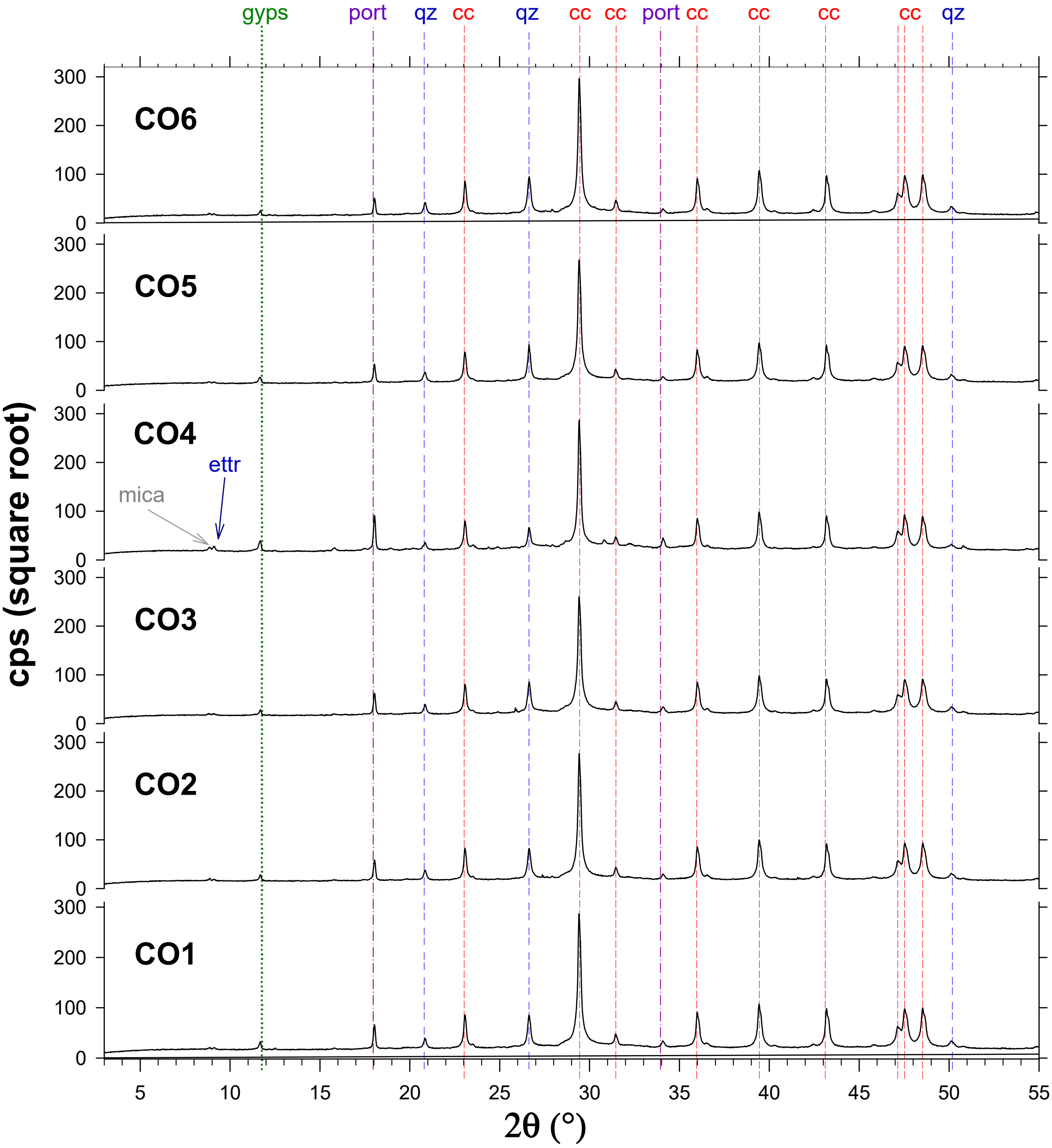
gyps
port
qz
cc
qz
cc
cc
port
cc
cc
cc
cc
qz
CO6
CO5
CO4
CO3
CO2
CO1
ettr
mica
cps (square root)
2θ (°)
0
100
200
300
5
10
15
20
25
30
35
40
45
50
55

(b)

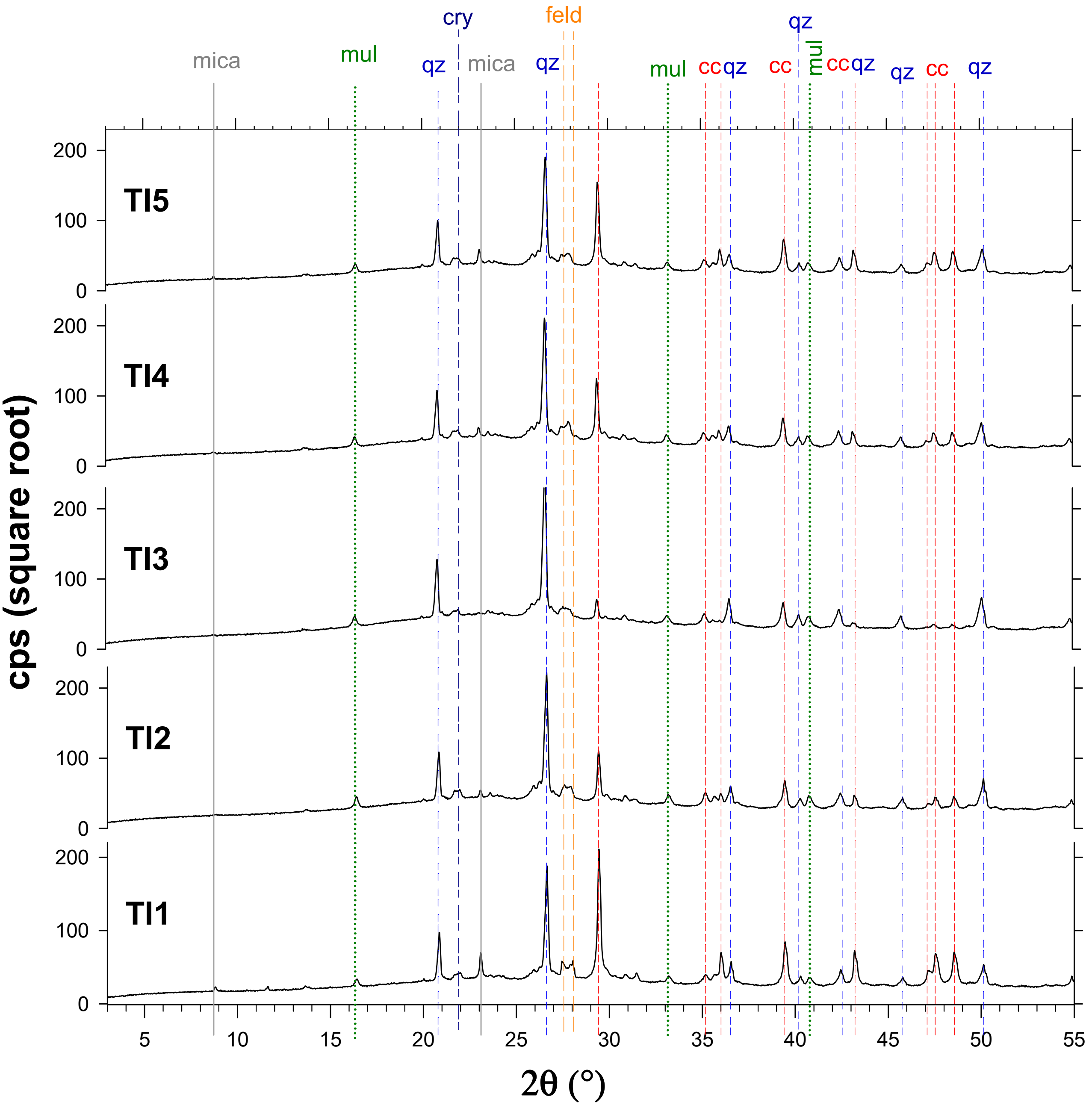
cry
feld
qz
mica
mul
qz
mica
qz
mul
cc
qz
cc
mul
cc
qz
qz
cc
qz
TI5
TI4
TI3
TI2
TI1
cps (square root)
2θ (°)
(c)

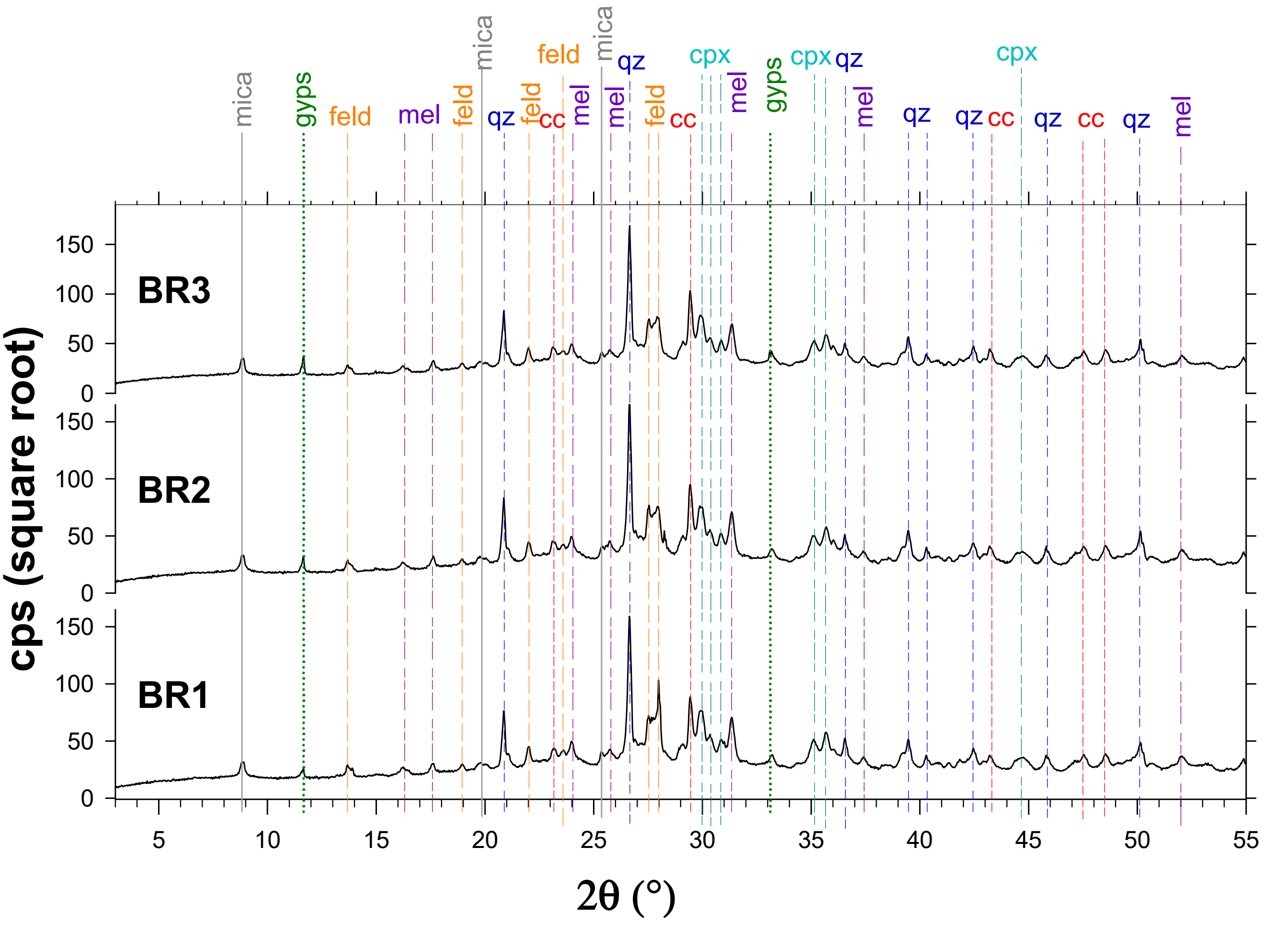


(d)

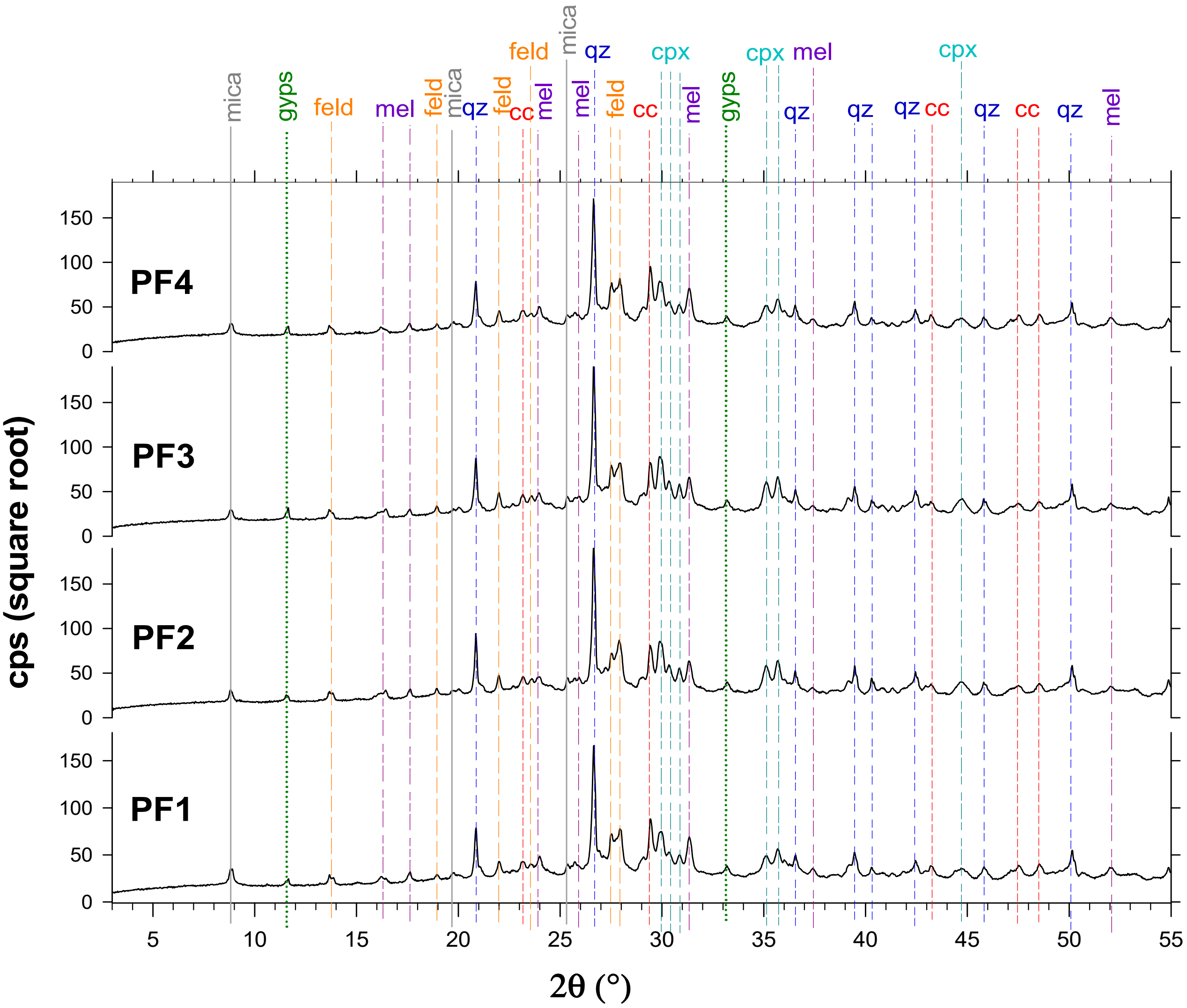
mica
gyps
feld
mel
feld
mica
qz
feld
cc
feld
mel
mica
mel
qz
feld
cc
cpx
mel
gyps
cpx
mel
qz
qz
qz
cc
cpx
qz
cc
qz
mel
PF4
PF3
PF2
PF1
cps (square root)
2θ (°)

(e)

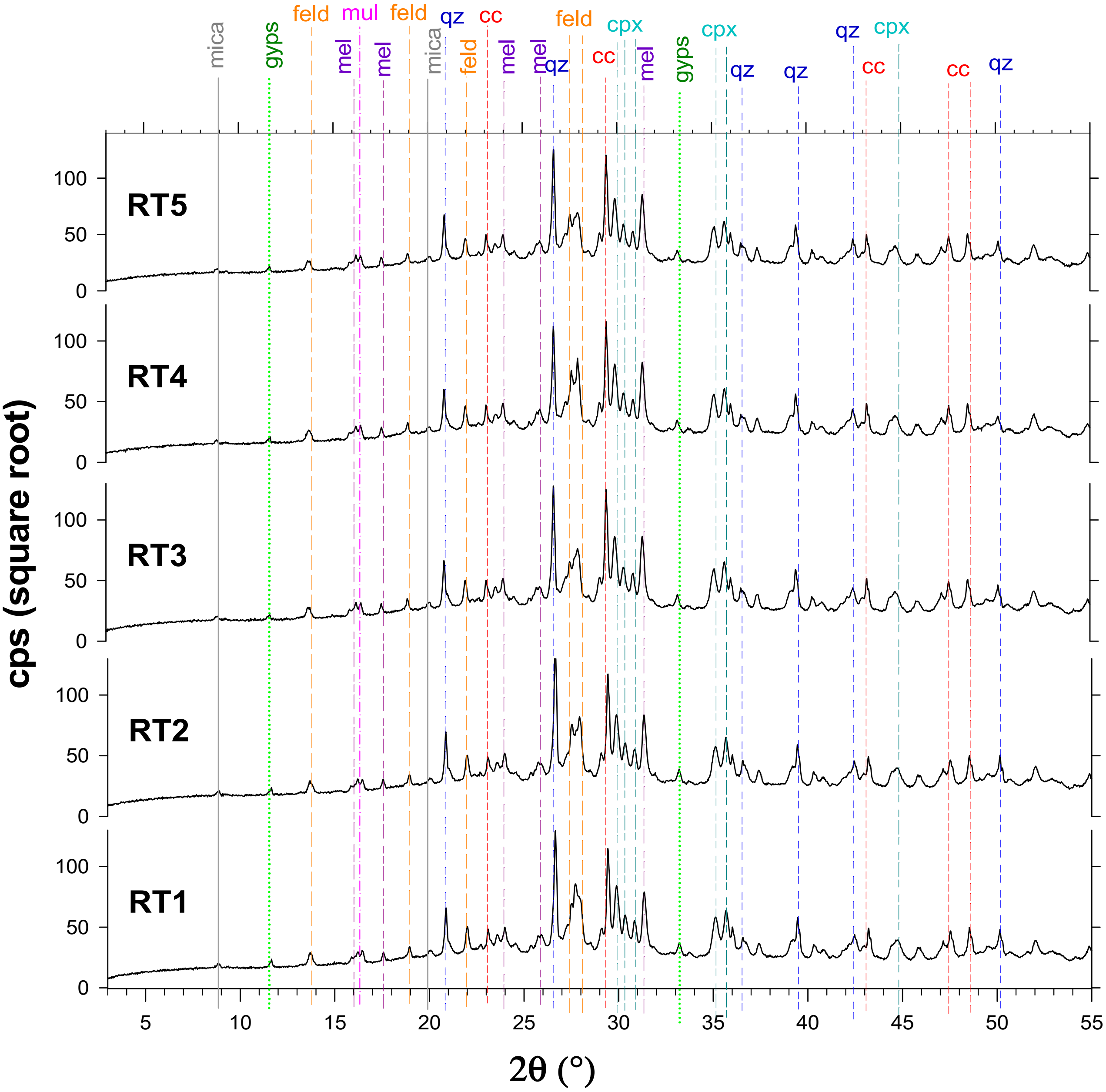


(f)

Figure S4. (a) (to be deposited as supplementary material). Stacked XRPD patterns from 3 to 55 of 2θ° (the actual scans are from 2 to 90 of 2θ°) for the five RA-NS-100 samples corresponding to CDW RA sorted as natural stones (Tables 2 and 3). The vertical lines correspond to the most intense Bragg reflections of crystalline standards, cps indicates counts per second and acronyms of crystalline phases as reported in Table 3, except feld (feldspars) that indicates either anorthite, albite and/or orthoclase. (coloured);

(b) (to be deposited as supplementary material). Stacked XRPD patterns from 3 to 55 of 2θ° (the ac-

tual scans are from 2 to 90 of 2θ°) for the six RA-CO-100 samples corresponding to CDW RA sorted as concrete and mortars (Tables 2 and 3). The vertical lines correspond to the most intense Bragg reflections of crystalline standards, cps indicates counts per second and acronyms of crystalline phases as reported in Table 3.

(c) (to be deposited as supplementary material). Stacked XRPD patterns from 3 to 55 of 2θ° (the actual scans are from 2 to 90 of 2θ°) for the five RA-TI-100 samples corresponding to CDW RA sorted as tiles (Tables 2 and 3). The vertical lines correspond to the most intense Bragg reflections of crystalline standards, cps indicates counts per second and acronyms of crystalline phases as reported in Table 3, except feld (feldspars) that indicates either anort, alb and/or orth.

(d) (to be deposited as supplementary material). Stacked XRPD patterns from 3 to 55 of 2θ° (the actual scans are from 2 to 90 of 2θ°) for the three RA-BR-100 samples corresponding to CDW RA sorted as bricks (Tables 2 and 3). The vertical lines correspond to the most intense Bragg reflections of crystalline standards, cps indicates counts per second and acronyms of crystalline phases as reported in Table 3, except feld (feldspars) that indicates either anort, alb and/or orth.

(e) (to be deposited as supplementary material). Stacked XRPD patterns from 3 to 55 of 2θ° (the actual scans are from 2 to 90 of 2θ°) for the three RA-PF-100 samples corresponding to CDW RA sorted as perforated bricks (Tables 2 and 3). The vertical lines correspond to the most intense Bragg reflections of crystalline standards, cps indicates counts per second and acronyms of crystalline phases as reported in Table 3, except feld (feldspars) that indicates either anort, alb and/or orth.

(f) (to be deposited as supplementary material). Stacked XRPD patterns from 3 to 55 of 2θ° (the actual scans are from 2 to 90 of 2θ°) for the three RA-RT-100 samples corresponding to CDW RA sorted as roof tiles (Tables 2 and 3). The vertical lines correspond to the most intense Bragg reflections of crystalline standards, cps indicates counts per second and acronyms of crystalline phases as reported in Table 3, except feld (feldspars) that indicates either anort, alb and/or orth.
